# DNA‐Loaded Nanoparticles Reprogram the Tumor Immune Microenvironment to Treat Brain Tumors

**DOI:** 10.1002/smsc.202500475

**Published:** 2026-01-05

**Authors:** Joanna Yang, Divyaansh Raj, Hasan Slika, Aanya Shahani, Leonardo Cheng, Manav Jain, Ethan Idnani, Kathryn M. Luly, FNU Ruchika, Caitlin Kraft, Charles Eberhart, Henry Brem, Betty Tyler, Jordan J. Green, Stephany Y. Tzeng

**Affiliations:** ^1^ Department of Biomedical Engineering Johns Hopkins University School of Medicine Baltimore MD 21231 USA; ^2^ Translational ImmunoEngineering Center, Translational Tissue Engineering Center Johns Hopkins University School of Medicine Baltimore MD 21231 USA; ^3^ Department of Neurosurgery Johns Hopkins University School of Medicine Baltimore MD 21205 USA; ^4^ Department of Pathology Johns Hopkins University School of Medicine Baltimore MD 21231 USA; ^5^ Department of Oncology and the Sidney Kimmel Comprehensive Cancer Center Johns Hopkins University School of Medicine Baltimore MD 21231 USA; ^6^ Department of Ophthalmology Johns Hopkins University School of Medicine Baltimore MD 21231 USA; ^7^ Departments of Materials Science & Engineering and Chemical & Biomolecular Engineering Johns Hopkins University Baltimore MD 21218 USA; ^8^ Institute for NanoBioTechnology and the Bloomberg∼Kimmel Institute for Cancer Immunotherapy Johns Hopkins University Baltimore MD 21231 USA

**Keywords:** cytotoxic T cells, glioblastoma, immunotherapy and immunoengineering, meningioma, nonviral gene therapy

## Abstract

Despite advances in treatment and therapeutic strategies, patients with brain tumors, including glioblastoma (GBM) and meningioma, still face high rates of recurrence, morbidity, and mortality. Nonviral biodegradable nanoparticles are advanced materials with the potential to reprogram brain tumor cells and the tumor immune microenvironment. Localized delivery of poly(beta‐amino ester) nanoparticles encapsulating immunostimulatory genes is utilized to reprogram brain tumor cells into tumor‐associated antigen‐presenting cells (tAPCs) by inducing overexpression of costimulatory 4‐1BBL on the surface of brain tumor cells and IL‐12 secreted into the tumor microenvironment. In both a humanized mouse model using human meningioma (IOMM‐Lee) and an immunocompetent syngeneic orthotopic model using mouse GBM (CT‐2A), delivery of 4‐1BBL/IL‐12 DNA‐loaded nanoparticles results in reduced tumor growth, as well as complete tumor regression and long‐term survival in some animals. The 4‐1BBL/IL‐12 gene delivery platform is an antigen‐agnostic, off‐the‐shelf biotechnology that can successfully activate cytotoxic T‐cells in tumors, improve tumor infiltration by immune cells, and enhance antitumor responses to otherwise refractory brain tumors. This nanoparticle reprogramming approach can lead to safe, long‐lasting endogenous cellular immune responses that specifically target multiple types of brain tumors that exhibit antigen heterogeneity in a patient‐accessible manner without using viruses or ex vivo cellular manufacturing.

## Graphical abstract

Biodegradable poly(beta‐amino ester) nanoparticles can be employed to deliver immunostimulatory genes (4‐1BBL and IL‐12) to reprogram brain tumor cells into antigen‐presenting cells to stimulate an anti‐tumor response, resulting in tumor regression and long‐term survival in GBM and meningioma models. This platform represents an antigen‐agnostic strategy that can lead to a clinically accessible platform for personalized immunotherapy in brain cancer.

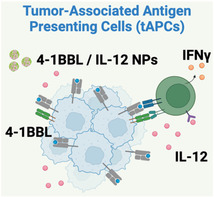

## 1 Introduction

Nearly 85 000 individuals are diagnosed with primary brain tumors each year in the United States.^[^ ^1^ ^]^ Glioblastoma (GBM) and meningioma represent the most common malignant and nonmalignant primary central nervous system (CNS) tumors, respectively. Despite meaningful advances in surgical technique, chemotherapy, and radiation therapy, GBMs remain refractory to treatment, while grade II and grade III meningiomas have an unacceptably high recurrence rate. GBM remains the most aggressive CNS tumor with 1‐year and 5‐year survival rates of around 42.9% and 6.9%.^[^ ^2^ ^]^ Meanwhile, grade II and grade III meningiomas demonstrate a recurrence rate of nearly 29%–94%.^[^ ^3^, ^4^, ^5^ ^]^ These tumors codevelop with their tumor immune microenvironment to not only escape the host response through an immunosuppressive state but also induce tumor stem cell (TSC) plasticity and self‐renewal.^[^ ^6^ ^]^ Together, these characteristics—alongside a high mutational burden and genetic instability—enhance the resilience of GBM and recurrent meningioma against targeted treatment strategies.

The World Health Organization recently reclassified primary brain tumors using novel genetic and molecular markers to further stratify them into distinct subtypes.^[^ ^5^ ^]^ Genetic and molecular analysis of GBM and recurrent meningioma tumors reveals heterogeneous cell populations and an immunosuppressive microenvironment that position immunotherapy as an attractive alternative strategy. Prior work has shown that a scarcity of invading T cells, reduction of microglial cytokine expression, and increased expression of PDL1 help tumor cells escape host immune response.^[^ ^7^ ^]^ To target this mechanism, dendritic cell vaccines, checkpoint inhibitors, CAR T‐cell therapy, and nanoparticle‐mediated delivery of neo‐antigen vaccines or suicide genes have emerged as potential candidates. These strategies, however, have faced several challenges and shown limited efficacy.^[^ ^8^, ^9^, ^10^, ^11^, ^12^ ^]^ Chiefly, these strategies require targeting of or inducing a ubiquitous antigen in a patient's tumor. Furthermore, these therapies require a priori knowledge of the specific tumor antigens present in heterogeneous TSC populations that evolve with treatment, presenting a moving target that further complicates their application.^[^ ^13^ ^]^

An alternative strategy involves utilizing gene therapy to reprogram tumor cells to express genes complementary to the host endogenous adaptive immune response without selectively targeting predetermined tumor antigens. Intracellular delivery of such genes can be achieved by complexing them with a synthetic polymer to fabricate nanoparticles. Synthetic polymeric nanoparticles have been established as a promising delivery modality due to their low toxicity, low immunogenicity, effectiveness, and ease of scaled‐up manufacturing.^[^ ^14^ ^]^ Positively charged polymers and negatively charged nucleic acids are electrostatically self‐assembled to form a nanoparticle complex.^[^ ^15^ ^]^ This complex can then efficiently enter cells to deposit genetic cargo for programming and elicit a desired immune response. Among synthetic nanoparticles, ones based on poly(beta‐amino ester)s (PBAEs) offer improved efficacy^[^ ^16^ ^]^ and quick hydrolytic biodegradability^[^ ^17^ ^]^ compared to many other nanoparticle systems, and improved safety and cargo capacity compared to leading viral systems.^[^ ^18^ ^]^ Previous research has shown PBAE nanoparticles to exhibit a natural tropism toward cancer cells over healthy astrocytes, making them a favorable gene delivery vehicle for meningioma and GBM.^[^ ^4^, ^19^ ^]^

We hypothesized that across multiple forms of brain cancer, the adaptive immune system could be activated and survival increased by inducing expression in the brain tumors of a costimulatory receptor and a secreted cytokine signal. T cells require activation through Signal 1, antigen presented on major histocompatibility complex (MHC); Signal 2, a costimulatory receptor; and Signal 3, a cytokine signal. The proposed gene therapy strategy is designed to induce the expression of Signal 2 and Signal 3 and leverages endogenous MHC I expression as Signal 1 to reprogram brain tumor cells into tumor antigen‐presenting cells (tAPCs). Crucially, this approach circumvents the need for prior knowledge of a specific, targetable antigen profile.

Recent research has identified costimulatory (Signal 2) receptors such as 4‐1BBL and cytokine IL‐12 (Signal 3) to preferentially recruit natural killer and cytotoxic CD8+ cells and increase affinity to MHC I complexes on tAPCs.^[^ ^20^, ^21^ ^]^ Furthermore, PD‐L1 expression is increased in resistant/recurrent GBM and meningioma patient tumors and represents a possible adjuvant target to sustain the immune response. In this study, we employ PBAEs carrying DNA encoding 4‐1BBL and IL‐12 to reprogram brain tumors by evaluating their effect against human meningioma (IOMM‐Lee) and murine glioma cells (CT‐2A). The CT‐2A glioma cell line demonstrates several immunologic and genetic features that resemble TSC's in GBM and is suitable for orthotopic studies in mice.^[^ ^22^ ^]^ The IOMM‐Lee human cell line was isolated from a recurrent chemo‐ and radiation therapy‐resistant patient sample and requires studies in humanized mice. This work aims to develop and validate a tumor immune microenvironment reprogramming strategy to treat various brain tumor types, as well as evaluate specific PBAE nanoparticles for their efficacy and safety as nonviral gene delivery agents suitable for brain tumor immunotherapy.

## 2 Results

### 2.1 Nanoparticle Formulations and in vitro Characterization

**Figure 1:**
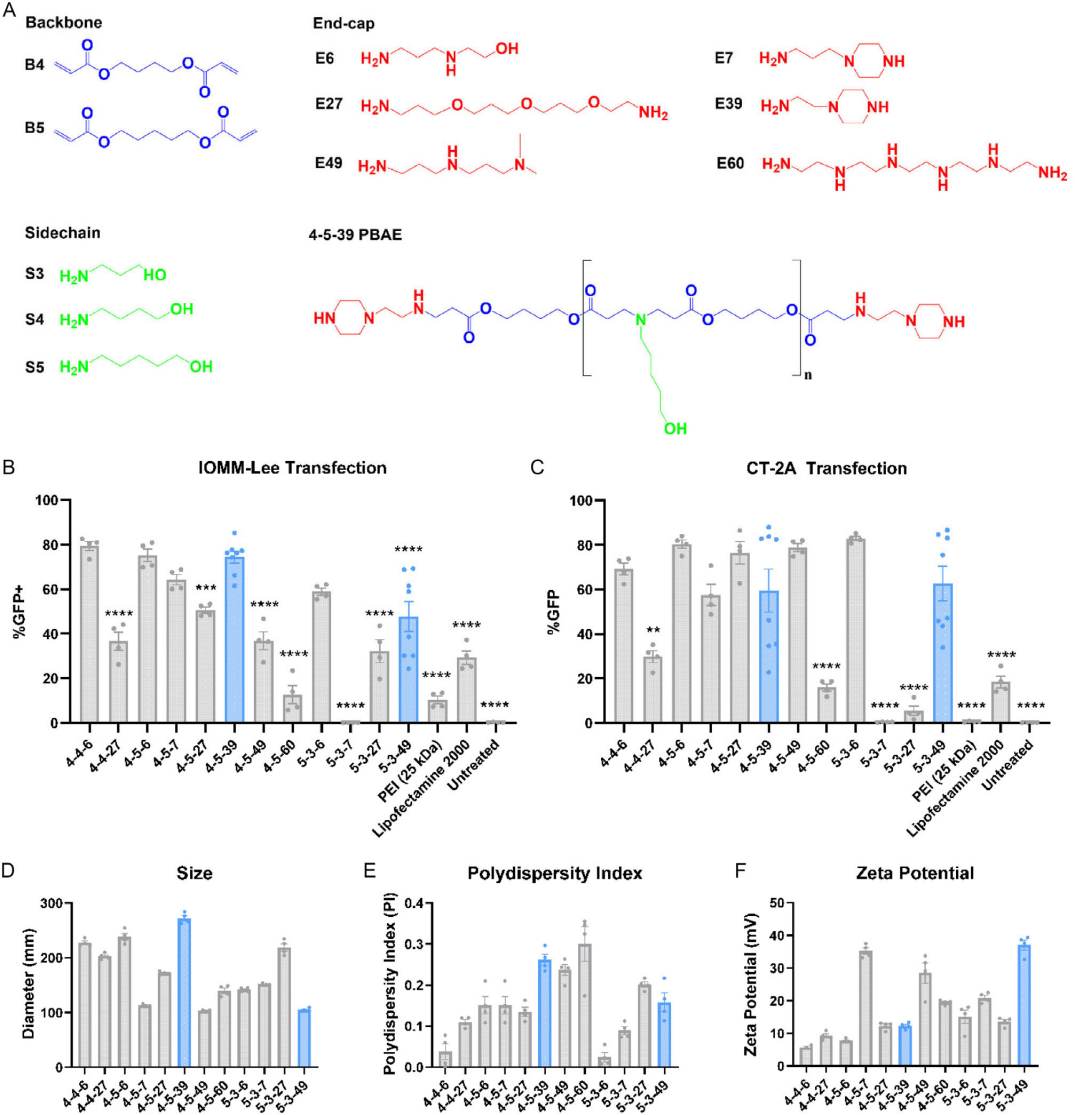
PBAE nanoparticle formulations transfect IOMM‐Lee cells and CT‐2A cells. A) Backbone, sidechain, and end‐cap monomers used to form PBAEs 4‐5‐39 and 5‐3‐49 are depicted, along with other monomers tested. The complete structure for 4‐5‐39 is also shown. B) IOMM‐Lee transfection with PBAE nanoparticles delivering 600 ng of DNA at 60 w/w (one‐way ANOVA with posthoc Dunnett's test, compared to 4‐5‐39). C) CT‐2A transfection with PBAE nanoparticles delivering 600 ng of DNA at 60 w/w (one‐way ANOVA with post hoc Dunnett's test, compared to 5‐3‐49). D–F) Diameter, polydispersity index, and zeta potential of PBAE nanoparticles tested in (B) and (C) as measured by DLS and electrophoretic mobility. Significance is represented by *P ≤ 0.05, **P ≤ 0.01, ***P ≤ 0.001, and ****P ≤ 0.0001. Each data bar represents mean ± SEM with four technical replicates unless otherwise indicated. 4‐5‐39 and 5‐3‐49 data bars in (B) and (C) represent mean ± SEM with eight technical replicates.

To evaluate the potential for efficient nonviral gene delivery to IOMM‐Lee and CT‐2A cells, candidate gene delivery structures based on our lab's previous investigations were identified.^[^ ^20^, ^23^, ^24^ ^]^ Twelve PBAE polymers, 4‐4‐6, 4‐4‐27, 4‐5‐6, 4‐5‐7, 4‐5‐39, 4‐5‐49, 4‐5‐60, 5‐3‐6, 5‐3‐7, 5‐3‐27, and 5‐3‐49, were synthesized via Michael Addition reaction from commercially available monomers (**Figure** **1**A, **Table** **1**). To validate gene delivery in vitro, IOMM‐Lee and CT‐2A cells were transfected with nanoparticles carrying 600 ng of green fluorescent protein (GFP) reporter plasmid at 60 w/w (weight/weight polymer to DNA ratio) in 20 μL of nanoparticles added to 100 μL of complete culture media (Figure 1B,C). Geometric mean fluorescence intensity of the GFP reporter gene, indicating transfection, and cell counts, a measure of viability, are further shown in Figure S1A,B, Supporting Information. The 4‐5‐39 nanoparticles caused 74 ± 3% transfection in IOMM‐Lee cells (Figure 1B), and 63 ± 8% in CT‐2A cells (Figure 1C). Cell counts following nanoparticle administration were also measured as a metric for toxicity (Figure S1C,D, Supporting Information).

**Table 1:** PBAE monomers.

	Monomer	Chemical name	Manufacturer	CAS no.
Backbone (B)	4	1, 4‐butanediol diacrylate	Alfa Aesar	1070‐70‐8
	5	1,4‐pentanediol diacrylate	Monomer‐Polymer and Dajac Labs	36 840‐85‐4
Sidechain (S)	3	3‐amino‐1propanol	Alfa Aesar	156‐87‐6
	4	4‐amino‐1butanol	Fisher Scientific	13 325‐10‐05
	5	5‐amino‐1pentanol	Alfa Aesar	2508‐29‐4
Endcap (E)	6	2‐(3‐aminopropylamino)ethanol	Sigma Aldrich	4461‐39‐6
	7	1‐(3‐aminopropyl)‐4‐methylpeperazine	Alfa Aesar	4572‐031
	27	4,7,10‐trioxa‐1,13‐tridecanediamine	Sigma Aldrich	4246‐51‐9
	39	1‐(2‐aminoethyl)piperazine	Alfa Aesar	140‐31‐8
	49	N,N‐dimethyldipropylenetriamine	Sigma Aldrich	1056‐329‐8
	60	Pentaethylenehexamine	Santa Cruz Biotechnology	4067‐16‐7

Dynamic light scattering (DLS) was also used to measure the hydrodynamic particle diameters of the PBAE nanoparticles and electrophoretic mobility was evaluated to measure surface charge, measured as zeta potential, for the nanoparticles. All nanoparticles were formulated at a 60:1 polymer to DNA w/w with 600 ng of DNA in 20 μL of nanoparticles (Figure 1D–F). 4‐5‐39 nanoparticles were 272 ± 5 nm (mean ± SEM in diameter with an average zeta potential of 12 ± 1 mV, while 5‐3‐49 nanoparticles were 105 ± 2 nm in diameter with an average zeta potential of 37 ± 2 mV.

4‐5‐39 was selected for further testing as it demonstrated high transfection efficiency in IOMM‐Lee and CT‐2A cells via both percent of cells transfected and mean fluorescence intensity of GFP expressed, while maintaining high cell viability in both cell lines. 5‐3‐49 was also selected for further testing as it retained high transfection efficiency in CT‐2A cells but offered different physical characteristics, such as smaller in size and higher zeta potential.

Delivery efficiency of 4‐5‐39 nanoparticles was compared to that of Lipofectamine 2000 and 25 kDa polyethylenimine (PEI), both of which are industry standards for transfection. In IOMM‐Lee cells, 4‐5‐39 nanoparticles demonstrated significantly higher transfection compared to both PEI (*p* < 0.0001) and Lipofectamine (*p* < 0.0001) (Figure 1B and S2A, Supporting Information). Likewise, in CT‐2A cells, 5‐3‐49 nanoparticles also demonstrated significantly higher transfection than PEI (*p* < 0.01) and Lipofectamine (*p* < 0.0001) (Figure 1C and S2B, Supporting Information). Cell viability in both cell lines between transfection vehicles was assessed by measuring metabolic activity in cells, normalized to cells that received no treatment (Figure S2C,D, Supporting Information). In IOMM‐Lee cells, 4‐5‐39 nanoparticles exhibited higher cell viability (81 ± 4%) compared to Lipofectamine 2000 (68 ± 3 %), but lower compared to PEI (99 ± 2%). In CT‐2A cells, 5‐3‐49 nanoparticles exhibited 74 ± 4% cell viability compared to Lipofectamine 2000 (96 ± 4%) and PEI (107 ± 6%). Despite some reductions in cell viability, transfection delivery was much higher with PBAE nanoparticles and thus they were employed as the gene delivery vehicle of choice.

Encapsulation efficiency for 4‐5‐39 nanoparticles was 100 ± 1% pre and postfreeze/thaw, and encapsulation efficiency for 5‐3‐49 nanoparticles made fresh was 97 ± 1% pre and postfreeze/thaw. Notably, encapsulation efficiency for both particles was the same before and after storage at −80 °C. Degradation properties of the 4‐5‐39 PBAE were measured after incubation in phosphate‐buffered saline (PBS) at pH 7, and increased incubation time correlated with decreasing M_n_ and M_w_ (Figure S3, Supporting Information and **Table** **2**). The calculated half life of the 4‐5‐39 PBAE in pH 7 was 2 h (Figure S3, Supporting Information).

**Table 2:** 4‐5‐39 polymer degradation.

Hour	M_n_ [Da]	M_w_ [Da]	PD
0	2614.333	19297.67	7.39273
1	1204.333	11998.67	9.979285
2	758	9446	12.46104
4	543.6667	6025.333	11.08553
8	461	574.6667	1.254079
24	304.6667	408.3333	1.343593

### 2.2 Delivery of 4‐5‐39 Nanoparticles to IOMM‐Lee Cocultured with Healthy Human Astrocytes

To investigate PBAE tropism for brain cancer cells over healthy cells,^[^ ^4^, ^25^ ^]^ we evaluated the selectivity of 4‐4‐6, 4‐4‐27, and 4‐5‐39 for IOMM‐Lee meningioma cells compared to human astrocytes. IOMM‐Lee cells, engineered to stably express tdTomato, and human astrocytes were plated together and transfected with GFP‐loaded nanoparticles. We assessed plating ratios of 10:1, 5:1, and 1:1 IOMM‐Lee cells to astrocytes prior to transfection. Interestingly, when cells IOMM‐Lee cells were plated at a 1:1 ratio with the astrocytes, the majority of cells harvested three days after plating were astrocytes. Percentages of IOMM‐Lee cells and human astrocytes after culturing at a 10:1 ratio are shown 48 h after transfection (Figure S4A, Supporting Information). While average transfection in IOMM‐Lee cells cultured at a 10:1 ratio with healthy astrocytes appeared higher than that of healthy astrocyte cells, and certain 4‐4‐6 nanoparticles demonstrated significantly higher transfection in tumor versus healthy cells, ultimately, most particles did not demonstrate a significant tropism towards the meningioma cells (Figure S4B, Supporting Information). Fluorescence microscopy images are shown in Figure S4C, Supporting Information. Cell ratios, transfection results, and fluorescent microscopy for the 5:1 and 1:1 ratio can be found in Figure S5, Supporting Information.

### 2.3 In Vitro Expression of Immunostimulatory Signals

**Figure 2:**
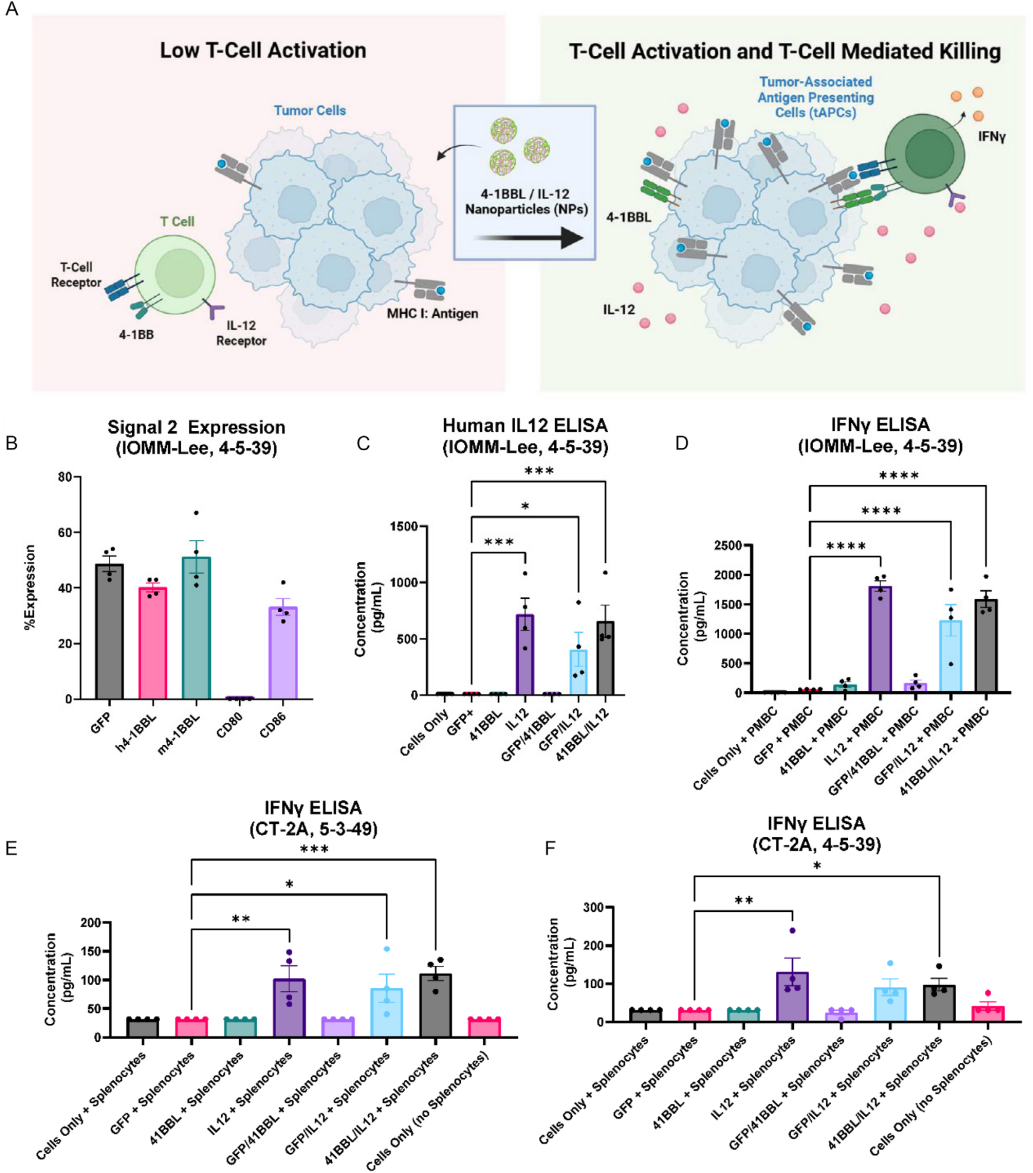
In vitro transfection with reprogramming PBAE nanoparticles demonstrates expression of T‐cell activation cytokines and inflammation markers. A) Delivery of Signal 2 (4‐1BBL) and Signal 3 (IL‐12) can lead to tumor reprogramming into tumor‐associated antigen‐presenting cells. B) 4‐5‐39 nanoparticles successfully transfect IOMM‐Lee cells with a variety of Signal 2s, including 4‐1BBL, CD80, and CD86. C) Delivery of IL‐12 (Signal 3) alongside 4‐1BBL and GFP both demonstrate IL‐12 expression. Despite the IL‐12 condition containing double the amount of IL‐12 plasmid, there was no significant difference between the IL‐12 and the 4‐1BBL/IL‐12 conditions (one‐way ANOVA, Dunnett's test, compared to GFP). D) Codelivery of 4‐1BBL and IL‐12 in IOMM‐Lee cells results in significantly higher expression of inflammatory marker IFN‐γ compared to delivery of only GFP, suggesting the presence of an immune response (one‐way ANOVA, Dunnett's test, compared to GFP condition). E–F) Codelivery of 4‐1BBL and IL‐12 in CT‐2A cells also results in significantly higher expression of IFN‐γ with both 5‐3‐49 and 4‐5‐39 nanoparticles (5‐3‐49, *p* = 0.0009; 4‐5‐49, *p* = 0.0484) (one‐way ANOVA, Dunnett's test, compared to GFP condition). Significance is represented by *P ≤ 0.05, **P ≤ 0.01, ***P ≤ 0.001, and ****P ≤ 0.0001. Each data bar represents mean ± SEM with four technical replicates.

Costimulatory signal (Signal 2) and cytokine (Signal 3) are both required to induce tumor reprogramming into antigen‐presenting cells (**Figure** **2**A). To evaluate the appropriate costimulatory signal and cytokine for expression, we delivered Signal 2 genes 4‐1BBL, CD80, and CD86 to IOMM‐Lee cells using 4‐5‐39 nanoparticles. Expression levels were measured by flow cytometry. The percent of cells expressing 4‐1BBL after transfection was similar to transfection levels of the reporter gene (GFP), and 4‐1BBL was selected as the Signal 2 of choice (Figure 2B). Next, we evaluated the codelivery of 4‐1BBL and IL‐12 in cocultured transfected IOMM‐Lee cells with human peripheral blood mononuclear cells (PBMCs) to measure IL‐12 as well as interferon (IFN)‐gamma expression as a proxy for T‐cell activation. IL‐12 concentrations were significantly higher in the 4‐1BBL/IL‐12 tAPC condition compared to the GFP control condition (*p* = 0.0005) (Figure 2C). Additionally, although the IL‐12‐only group (600 ng IL‐12) was loaded with twice the IL‐12 DNA loaded in the 4‐1BBL/IL‐12 group (300 ng 4‐1BBL, 300 ng IL‐12), there was no significant difference in IL‐12 measured by ELISA. Lastly, the IFN‐gamma ELISA demonstrated that significantly more IFN‐gamma was produced in the 4‐1BBL/IL‐12 than in the untreated or 4‐1BBL‐only groups (Figure 2D). We then similarly evaluated IFN‐gamma expression in CT‐2A cells. Cells were transfected with both 4‐5‐39 and 5‐3‐49 PBAE nanoparticles after seeding. Cells treated with both 4‐1BBL and IL‐12 demonstrated significantly higher IFN‐gamma levels after delivery of either type of nanoparticle compared to the control (Figure 2 E,F).

### 2.4 Ex Vivo Tumor‐Killing

**Figure 3:**
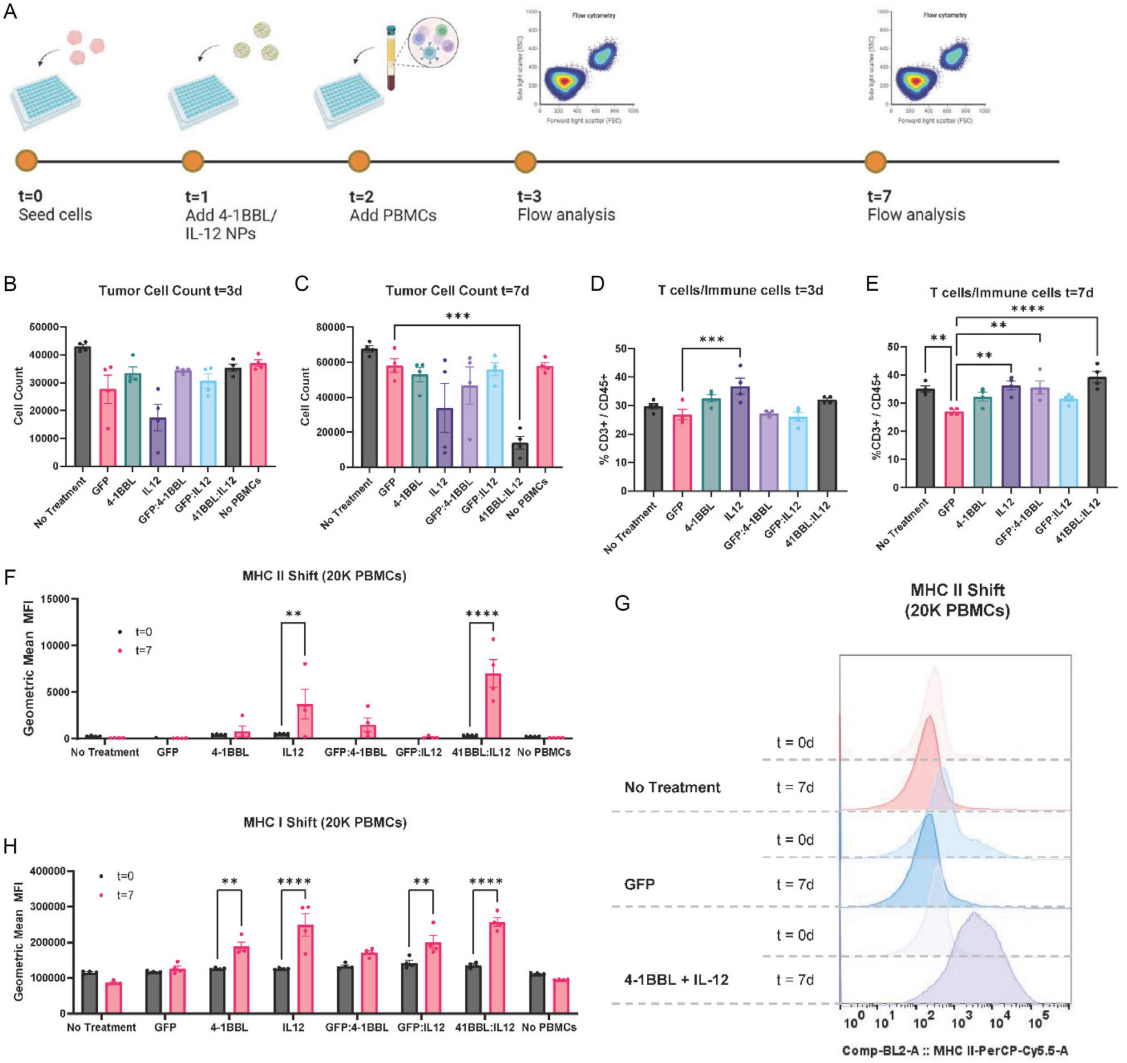
Ex vivo coculture of IOMM‐Lee cells with human PBMCs results in tumor cell death and a shift in MHC I and II expression. A) Experimental timeline reprogramming tumor cells and coculturing with PBMCs. B,C) Tumor cells were counted after 3 and 7 days. On the 7th day, tumor cell count was significantly reduced in groups treated with 4‐1BBL and IL‐12 (one‐way ANOVA, Dunnett's test, compared to GFP condition). D,E) Ratio of T‐cells to immune cells 3 days and 7 days after PBMC addition is depicted. There was an increase of T cell population relative to all other immune cells in the group treated with 4‐1BBL/IL‐12 nanoparticles compared to the GFP nanoparticles (one‐way ANOVA, Dunnett's test, compared to GFP condition). F,G) In treatment groups with IL‐12 or 4‐1BBL/IL‐12, there is a significant shift in MHC II expression after 7 days (two‐way ANOVA, Sidak's post‐test). H) There is a similar shift in MHC I expression after 7 days, with groups receiving IL‐12 or 4‐1BBL/IL‐12 nanoparticles exhibiting the most significant shift. Significance is represented by **P ≤ 0.01, ***P ≤ 0.001, and ****P ≤ 0.0001. Each data bar includes means ± standard error of the mean with four technical replicates.

IOMM‐Lee is a human patient‐derived cell line that requires a humanized mouse to evaluate treatment response. To validate tumor killing by T cells, we conducted an ex vivo experiment to evaluate tumor reprogramming and T‐cell response (**Figure** **3**A). Tumor cells were plated and transfected with 4‐5‐39 nanoparticles containing GFP, 4‐1BBL, or IL‐12. 24 h after transfection, IOMM‐Lee cells were cocultured with either 20 or 50 K human PBMCs and monitored for changes in cell count. On Day 7 for the 20 K condition, the cocultures treated with 4‐1BBL/IL‐12 had fewer tumor cells compared to those treated with GFP, ∼75% tumor‐cell killing (20 K PBMC; *p* = 0.0008) (Figure 3B,C). Tumor cell counts and the percentage of T cells among the immune cells for the conditions with 50 K PBMCs can be found in Figure S6A–D, Supporting Information. Cells were stained for CD45 and CD3 to identify T cells from other lymphocytes, macrophages, and dendritic cells (Figure 3D,E). On Day 7, the percentage of T cells out of all immune cells in the 4‐1BBL/IL‐12 group was significantly higher than in the GFP group (20 K PBMC; *p* < 0.0001). Cells were additionally stained for MHC I and II to measure productive antigen presentation to CD8+ and CD4 + T cells (Figure 3F–H, and Figure S6, E–G). In both the 50 K PBMC and 20 K PBMC conditions, there was a significant increase in both MHC I and MHC II expression per cell between Day 0 and Day 7 for cells treated with 4‐1BBL/IL‐12 nanoparticles (*p* < 0.0005).

### 2.5 In Vivo Transfection

#### 2.5.1 Heterotopic, Flank *In Vivo* Transfection

To validate the successful transfection of candidate nanoparticles in the in vivo setting, 4‐5‐39 and 5‐3‐49 were injected intratumorally in heterotopic models of CT‐2A and IOMM‐Lee. We began by implanting CT‐2A cells into the flanks of C57BL/6 mice and IOMM‐Lee cells into the flanks of athymic nude mice. On Day 7, 4‐5‐39 (30 w/w, 0.2 mg mL^−1^) nanoparticles complexed with luciferase DNA were injected directly into the tumor, and In Vivo Imaging System (IVIS) imaging was conducted 6, 24, and 48 h after nanoparticle delivery (Figure S7A, Supporting Information). To confirm the transfection efficacy for the 5‐3‐49 nanoparticles, C57BL/6 mice implanted with CT‐2A murine glioma in the flank were injected intratumorally with luciferase DNA‐loaded 5‐3‐49 nanoparticles. Successful transfection occurred in 2 out of the 5 tumors (Figure S7B, Supporting Information). In vivo transfection was also verified using IOMM‐Lee cells in humanized mice (Hu PBMC NSG DKO MHC I/II mice) heterotopically with the 4‐5‐39 nanoparticles (Figure S7C, Supporting Information). Successful transfection occurred in 5 out of the 5 tumors.

#### 2.5.2 Orthotopic Intracranial *In Vivo* Transfection

To validate intracranial transfection, we inoculated athymic nude mice with IOMM‐Lee and C57BL/6 mice with CT‐2A cells to establish orthotopic tumors. *IOMM‐Lee:* On Day 7, mice received four different 4‐5‐39 nanoparticle formulations complexed with GFP and delivered via convection‐enhanced delivery (CED) (30 w/w at 0.1 mg mL^−1^, 0.2 mg mL^−1^, and 0.05 mg mL^−1^ and 15 w/w at 0.1 mg mL^−1^). After 18 h, mice were euthanized, and the tumor was isolated to measure total GFP expression via GFP ELISA. The 30 w/w, 0.2 mg mL^−1^ formulation showed low GFP expression, which may be due to high concentration leading to poorer nanoparticle distribution, whereas the three other formulations demonstrated a bimodal distribution of GFP expression, where each group contained mice that exhibited either higher or lower expression of GFP (Figure S7D, Supporting Information). *CT‐2A:* On Day 7, mice received six different nanoparticle formulations complexed with GFP and delivered via CED (4‐5‐7, 5‐3‐27, and 5‐3‐49 at either 0.1 mg mL^−1^ or 0.2 mg mL^−1^) to identify the best nanoparticle candidate. After 18 h, mice were euthanized, and the tumor was isolated to measure total GFP expression via GFP ELISA. 5‐3‐49 (30 w/w, 0.1 mg mL^−1^) demonstrated the highest GFP expression compared to all other conditions (Figure S7E, Supporting Information).

### 2.6 Heterotopic, Flank Model Survival Study

After demonstrating in vitro efficacy and validating in vivo transfection in CT‐2A and IOMM‐Lee cell lines, we proceeded to evaluate the in vivo therapeutic efficacy of the reprogramming strategy in a flank heterotopic model. The blood–brain barrier preserves the CNS as an immune‐privileged site, contributing significantly to the poor systemic immune response observed in CNS primary tumors. This heterotopic model serves as proof of concept for the gene therapy strategy.

#### 2.6.1 IOMM‐Lee Therapeutic Efficacy Study

**Figure 4:**
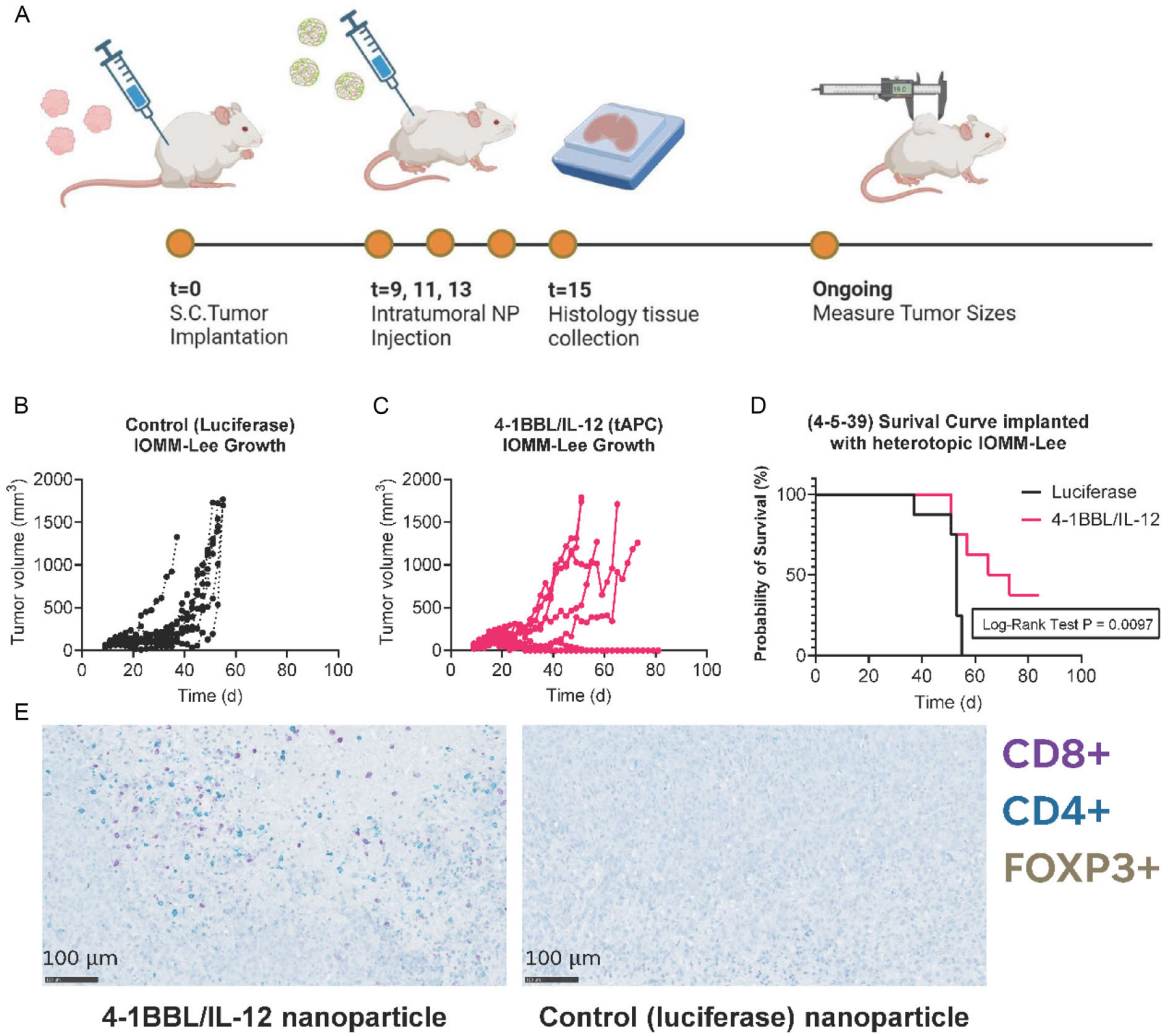
Reprogramming 4‐1BBL/IL‐12 PBAE nanoparticles demonstrate increased survival in an IOMM‐Lee meningioma humanized mouse model. A) Nanoparticle delivery schedule into heterotopic flank tumors. B,C) Growth of individual tumors was measured in the control (luciferase DNA) and 4‐1BBL/IL‐12 DNA group over 80 days. In the 4‐1BBL/IL‐12 DNA group, 3 mice had tumors that regressed completely. D) Survival curve of both groups shows that the mice treated with 4‐5‐39 PBAE 4‐1BBL/IL‐12 nanoparticles demonstrated significant survival (*p* = 0.0097, log rank test) compared to control mice treated with luciferase nanoparticles. There were three long term survivors (37.5%) in the group treated with 4‐1BBL/IL‐12 nanoparticles, compared to zero in the control group. **E**) IHC‐stained slides (20X) from the two treatment groups show T cell infiltration into the tumor of the mouse that received 4‐1BBL/IL‐12 nanoparticles. Human FOXP3+ cells were stained with DAB, human CD4+ cells were stained with teal, and human CD8+ cells are stained with purple.

First, to validate the use of this tumor model for long‐term studies, we implanted 1M or 2M IOMM‐Lee cells in the flank of each humanized mouse. Tumors continued to grow over 25 days, including after injection of 4‐5‐39 control nanoparticles carrying luciferase DNA (Figure S8, Supporting Information). To test treatment efficacy for meningioma, after 9 days, mice inoculated with 1M cells received intratumoral 4‐5‐39 nanoparticles according to the following groups: 1) 4‐1BBL/IL‐12 nanoparticles or 2) luciferase nanoparticles (**Figure** **4**A). Mice in the luciferase control group had a median survival of 53 days, while those treated with 4‐1BBL/IL‐12 showed significantly prolonged survival with a median survival of 69 days (*p* = 0.0097, log‐rank test) underscoring the efficacy of the tumor reprogramming approach (Figure 4B–D). Additionally, 37.5% of the mice receiving 4‐1BBL/IL‐12 experienced complete tumor regression and survived beyond 83 days, compared to 0 mice in the control group. Nanoparticle size, polydispersity index, and zeta potential were measured using DLS (Figure S9A–C, Supporting Information). Additional IHC staining for CD8+, CD4+, and FOXP3 + T cells demonstrated immune cell infiltration into mouse tumor that received 4‐1BBL/IL‐12 nanoparticles, and little immune cell infiltration into mouse tumor that received luciferase nanoparticles (Figure 4E). Images shown are at 20X, and additional images at 5X can be found in Figure S10, Supporting Information.

#### 2.6.2 CT‐2A Therapeutic Efficacy Study

**Figure 5:**
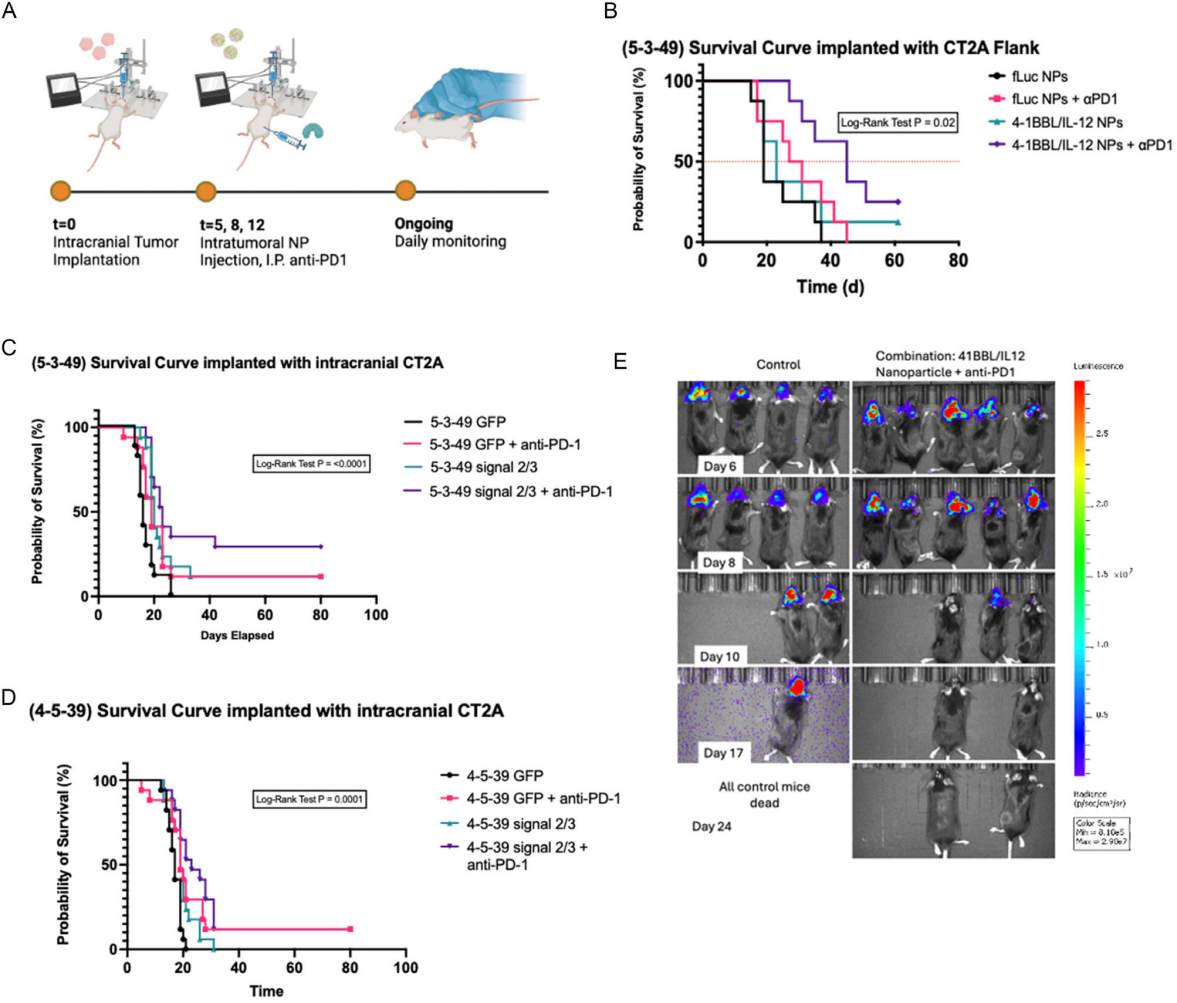
Reprogramming 4‐1BBL/IL‐12 PBAE nanoparticles demonstrate increased survival in orthotopic and flank glioma models. A) Nanoparticle injection schedule in intracranial tumor model. B) Kaplan–Meier curve showing the survival of C57BL/6 mice implanted with CT‐2A glioma cells in the flank showing significant increase in overall survival of mice that received an intratumoral injection of the 4‐1BBL/IL‐12 DNA‐loaded 5‐3‐49 nanoparticles and systemic anti‐PD‐1 therapy (p = 0.0097, log‐rank test). C,D) Kaplan–Meier curves showing the survival of C57BL/6 mice implanted intracranially with 75 000 CT‐2A glioma cells and treated with the 5‐3‐49 nanoparticle formulation (C) and the 4‐5‐39 nanoparticle formulation (D). The *p*‐values shown correspond to the log‐rank test of the overall survival distribution. E) Bioluminescent‐based imaging of C57BL/6 mice implanted with 125 000 firefly luciferase‐tagged CT‐2A glioma cells orthotopically and treated with control (intratumoral 5‐3‐49 GFP nanoparticles) or the combination therapy (intratumoral 5‐3‐49 4‐1BBL/IL‐12 nanoparticles and systemic anti‐PD‐1) showing complete resolution of previously established tumors in the combination group.

In the CT‐2A flank model, mice were similarly inoculated with CT‐2A cells. After 7 days, mice received intratumoral 5‐3‐49 nanoparticles according to the following groups: 1) 4‐1BBL/IL‐12 nanoparticles with systemically delivered anti‐PD‐1, 2) 4‐1BBL/IL‐12 nanoparticles only, 3) GFP nanoparticles with systemically delivered anti‐PD‐1, or 4) GFP nanoparticles only. At 7, 9, and 11 days postinoculation, the nanoparticles were intratumorally injected, coupled with systemic anti‐PD‐1 blockade therapy for the appropriate groups (**Figure** **5**A). Nanoparticle characteristics, including size, polydispersity index, and zeta potential, were assessed via DLS (Figure S9D–F, Supporting Information). While the improvement in median survival for the groups receiving monotherapy (4‐1BBL/IL‐12 or anti‐PD‐1) was not significant compared to control, the group receiving the anti‐PD‐1 and 4‐1BBL/IL‐12 combination therapy had an impressive statistically significant increase in median survival (*p* = 0.02) (Figure 5B). Notably, 25% of mice that received combination therapy and 10% of mice that received 4‐1BBL/IL‐12 monotherapy completely resolved their tumors by Day 60.

### 2.7 Orthotopic, Intracranial Survival Study

After validating that the reprogramming gene therapy strategy could evoke an immune response in the flank, we utilized an orthotopic, intracranial tumor model to evaluate IFN the treatment response was impacted by the unique tumor immune microenvironment of the brain. 160 Mice were inoculated with CT‐2A tumors and received three treatments of either the 5‐3‐49 or 4‐5‐39 nanoparticles. Mice were randomized into eight groups (*n* = 20) as follows: 1) 5‐3‐49 nanoparticles loaded with GFP DNA (control), 2) 5‐3‐49 nanoparticles loaded with GFP DNA + systemically delivered anti‐PD‐1, 3) 5‐3‐49 nanoparticles loaded with 4‐1BBL/IL‐12 DNA, 4) 5‐3‐49 nanoparticles loaded with 4‐1BBL/IL‐12 DNA + anti‐PD‐1, 5) 4‐5‐39 nanoparticles loaded with GFP DNA, 6) 4‐5‐39 nanoparticles loaded with GFP DNA + systemically delivered anti‐PD‐1, 7) 4‐5‐39 nanoparticles loaded with 4‐1BBL/IL‐12 DNA, and 8) 4‐5‐39 nanoparticles loaded with 4‐1BBL/IL‐12 DNA + systemically delivered anti‐PD‐1.

#### 2.7.1 5‐3‐49 Nanoparticles Results

The median survival in the combination group (group 4) was 23 days (95% confidence interval [CI], 20.0‐26.0) and in the monotherapy 4‐1BBL/IL‐12 group (group 3) was 20 days (95% CI, 18.7–21.3), which were both significantly greater than that for the control group (16 days [95% CI, 14.7–17.3]) (Figure 5C). The anti‐PD‐1 therapy alone did not significantly increase the median survival (19 days [95% CI, 16.3–21.7]) as compared to the control group. In the combination arm of this experiment, 35% of mice completely cleared their tumors, while 10% of mice that received 4‐1BBL/IL‐12 or anti‐PD‐1 monotherapy completely cleared the tumor and exhibited long‐term survival (>80 days). To further validate the ability of the treatment to achieve tumor clearance, this experiment was replicated in mice implanted with firefly luciferase‐tagged CT‐2A cells. The changes in tumor burden were followed by IVIS, which showed the ability of the combination therapy to result in resolution of previously established intracranial tumors in 25% of the mice (Figure 5E, Figure S11A, Supporting Information).

#### 2.7.2 4‐5‐39 Nanoparticles Results

Results from the 4‐5‐39 nanoparticles were similar to those observed with the 5‐3‐49 nanoparticles, with the anti‐PD‐1 monotherapy (group 6), 4‐1BBL/IL‐12 monotherapy (group 7), and the combination therapy (group 8) achieving a significant increase in overall survival as compared to the control (log‐rank *p*‐values: 0.001, 0.01, and 0.0001, respectively) (Figure 5D). In the combination arm and 4‐1BBL/IL‐12 monotherapy arm of this experiment, 10% of mice in each group achieved long‐term survival (>80 days).

### 2.8 Safety Assessment

The hematoxylin and eosin (H&E)‐stained sections of brains collected from the mice sacrificed 72 h after the last treatment were examined by an experienced neuropathologist (CE) to assess for any signs of treatment‐related toxicity or inflammation in a blinded manner. The neuropathologist noted signs of mild perivascular, periventricular, or leptomeningeal inflammation not adjacent to the tumor site in 2 out of 3 mouse brain samples in the group receiving the 5‐3‐49 nanoparticles loaded with 4‐1BBL/IL‐12 DNA (group 3), and in 3 out of 3 mouse brain samples in the groups receiving the 5‐3‐49 nanoparticles loaded with 4‐1BBL/IL‐12 DNA + anti‐PD‐1 (group 4) and 4‐5‐39 nanoparticles loaded with 4‐1BBL/IL‐12 DNA + systemically delivered anti‐PD‐1 (group 8). The examiner did not note any excessive inflammation or reactive gliosis or any alarming signs indicating toxicity.

### 2.9 Tumor Volume Assessment

Tumor volume was calculated based on H&E stained sections of brains collected from mice in the CT‐2A orthotopic survival study that were euthanized 72 h after the last treatment. The mean tumor volume for the combination treatment group was lower than the GFP nanoparticles (control) group in both the 5‐3‐49 and 4‐5‐39 cohorts. However, these differences did not reach statistical significance. Specifically, for the 5‐3‐49 cohort, the control group showed a mean tumor volume of 10.2 ± 9.7 mm^3^, whereas the combination treatment had a mean of 5.1 ± 6.9 mm^3^ (Figure S11B, Supporting Information). In the 4‐5‐39 group, the control group exhibited a mean tumor volume of 23 ± 16 mm^3^, compared to the combination treatment's mean of 7 ± 9 mm^3^ (Figure S11C, Supporting Information).

### 2.10 Immunohistochemistry for Immune Infiltration Assessment

**Figure 6:**
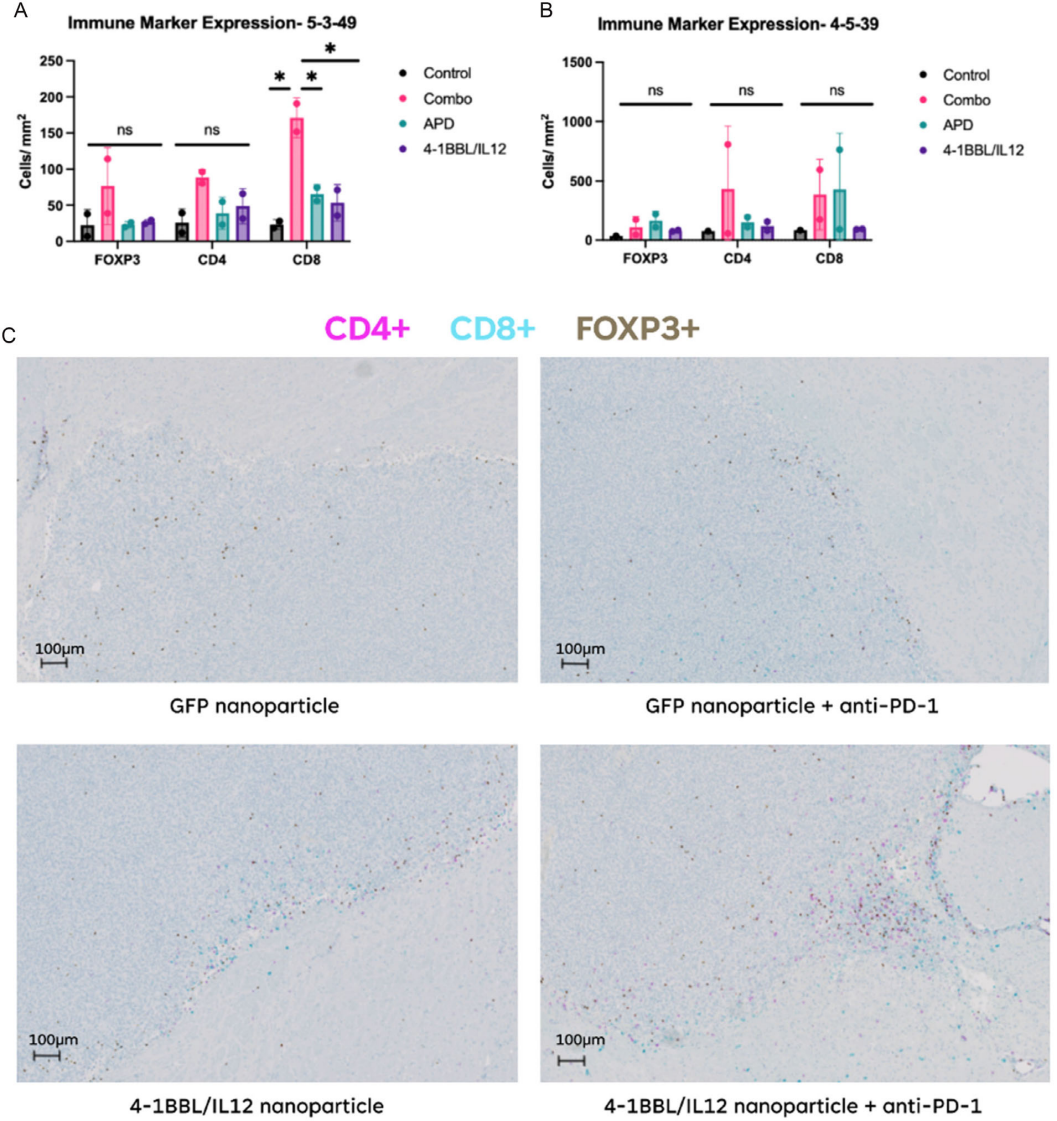
IHC (CD8, CD4, FOXP3) staining results assessing immune cell infiltration into orthotopically implanted CT‐2A tumors. A,B) Bar graph showing the number of positively stained cells/mm^2^ of tumor for each of the immune markers in mice treated with the 5‐3‐49 nanoparticle formulation (A) (*, *p*‐value of independent samples *t*‐test <0.05; ns, *p*‐value of independent samples *t*‐test ≥0.05) and 4‐5‐39 nanoparticle formulation (B). The graph depicts the individual counts for samples along with the median and interquartile range for each group. C) Sample images of IHC‐stained slides from each of the four treatment groups receiving the 5‐3‐49 nanoparticle formulation. FOXP3+ cells are stained with DAB, CD4+ cells are stained with pink, and CD8+ cells are stained with teal. The figure shows substantial immune cell infiltration into the center of the tumor in the mouse that received the 5‐3‐39 nanoparticles loaded with 4‐1BBL/IL‐12 DNA and systemic anti‐PD‐1 therapy.

To characterize the extent of immune‐cell activation during treatment, we stained tumor sections from mice euthanized in each group 72 h after the final treatment. Tissue sections were stained for FOXP3, CD4, and CD8. Immunohistochemistry (IHC) showed a significant increase in mean CD8 + T‐cell infiltration (*p* = 0.018) in the 5‐3‐49 combination therapy group (170 ± 30 cells per mm^2^) compared to the 5‐3‐49 control (23 ± 7 cells per mm^2^), along with an elevated CD4 cell infiltration, indicating robust immune activation in treated tumors (**Figure** **6**A,C). While CD4 and CD8 cell counts were elevated in the 5‐3‐49 monotherapy groups compared to the 5‐3‐49 control, these were not statistically significant (Figure 6A,C). Immune marker expression was not significantly different in FOXP3, CD4, and CD8 amongst the 4‐5‐39 nanoparticles groups (Figure 6B).

## 3 Conclusion

This study presents a novel approach to brain tumor immunotherapy, utilizing biodegradable PBAE nanoparticles to deliver DNA encoding the costimulatory molecule 4‐1BBL and the cytokine IL‐12 to brain tumors. The findings suggest that this strategy successfully reprograms tumor cells into tAPCs in vivo, facilitating the activation of adaptive immune responses and precipitating tumor cell killing. Furthermore, nanoparticle formulations fabricated from PBAEs 4‐5‐39 and 5‐3‐49 demonstrated successful reprogramming in both flank and orthotopic intracranial tumor models across two brain tumor subtypes, and also were efficacious in humanized mice, showing robustness of the overall approach in terms of both nanomaterials and brain tumor type. Notably, the combination of this gene therapy with checkpoint inhibition (anti‐PD‐1) resulted in improved survival outcomes, and in some cases, complete tumor regression. However, further studies in humanized and orthotopic models are needed to validate these observations and explore their broader translational potential.

The inability of checkpoint inhibitors to demonstrate a statistically significant clinical benefit in GBM clinical trials, despite promising outcomes in preclinical GBM models and clinical success in other solid tumors, underscores the profound immunosuppressive microenvironment characteristics of CNS tumors.^[^ ^26^, ^27^ ^]^ One of the most significant barriers to effective immunotherapy in CNS tumors is the heterogeneity of tumor antigens—both within individual tumors and across patient populations. Emerging strategies, such as dendritic cell vaccines and CAR‐T‐cell therapies, often depend on identifying and targeting specific antigens, which are known to vary significantly among patients.^[^ ^3^, ^28^, ^29^ ^]^ Moreover, the tumor microenvironment in GBM and meningioma is highly dynamic, with antigen profiles evolving over the course of chemotherapy and radiotherapy.^[^ ^30^, ^31^ ^]^ Consequently, there is an urgent need for immunotherapeutic strategies that do not rely on the stable presence of known, targetable antigens. Such an off‐the‐shelf strategy that does require patient‐specific tumor profiling and sequencing to generate patient‐specific tumor responses can also make treatment less expensive and more accessible to patients.

The approach investigated in the current study circumvents the challenge of antigen targeting by leveraging costimulatory (Signal 2) and cytokine (Signal 3) pathways to initiate a robust immune response without the need to preidentify tumor‐specific antigens. By inducing these signals, the nanoparticles allow broad T‐cell activation and infiltration, even in the presence of antigenic heterogeneity. This dynamic strategy could provide a more sustained immune response than current antigen‐dependent approaches, offering the potential for broader application across diverse CNS tumor types.

Previous studies using PBAE nanoparticles in CNS tumors have typically employed a cancer gene therapy strategy to deliver “suicide genes” aimed at selectively killing tumor cells.^[^ ^23^, ^24^ ^]^ While PBAE nanoparticles have shown some selectivity toward cancer cells in GBM and other malignancies, past research has not investigated tropism for meningioma, including IOMM‐Lee meningioma cell specificity over healthy astrocytes.^[^ ^4^ ^]^ A lack of selectivity presents a potential challenge when considering off‐target effects of cancer gene therapy, as the delivery of suicide genes and prodrugs to healthy cells could trigger apoptosis across multiple cell types in the brain. In contrast, the delivery of costimulatory and cytokine signals specifically to brain tumor cells avoids the need to directly kill tumor cells, and instead, reprograms them to enact a secondary endogenous cellular immune response. This pro‐inflammatory shift in the tumor microenvironment preserves neoantigen presentation via endogenous MHC I, thus potentially minimizing off‐target effects while maintaining robust immune‐mediated killing of brain tumors. Though no tropism towards the IOMM‐Lee meningioma cells over healthy astrocytes was observed with the 4‐5‐39 nanoparticle in vitro, some tropism is seen with the 4‐4‐6 nanoparticle formulation, indicating potential for targeting improvement in future work.

The independent roles of 4‐1BBL and IL‐12 in promoting immune activation have been well documented.^[^ ^4^, ^32^ ^]^ In the current study, their codelivery via PBAE nanoparticles resulted in significantly higher IFN‐gamma production compared to controls, highlighting their ability to effectively activate T cells. Immunological escape mechanisms in GBM and meningioma are characterized by low T‐cell infiltration and upregulation of PD‐L1 in tumor cells and peripheral macrophages.^[^ ^7^, ^33^, ^34^ ^]^ To counter these mechanisms, we combined the nanoparticle therapy with systemic delivery of anti‐PD‐1 antibody, aiming to enhance immune cell infiltration and differentiation within the tumor microenvironment. The results in the CT‐2A model, particularly the 35% tumor resolution rate in intracranial tumors, suggest that this combination may recruit and activate a stronger and more sustained immune response.

TSCs are among the most dynamic and resilient populations within tumors.^[^ ^6^, ^35^ ^]^ Their inherent plasticity is thought to drive resistance to conventional therapies and contribute to tumor recurrence.^[^ ^1^, ^36^ ^]^ In this study, the CT‐2A model—known to closely mimic glioma stem cells—and the IOMM‐Lee model, derived from a recurrent meningioma, were selected to represent these challenging tumor subpopulations.^[^ ^37^, ^38^, ^39^ ^]^ The observed survival benefit in both flank and intracranial models, particularly with the combination of anti‐PD‐1 and 4‐1BBL/IL‐12, suggests that this strategy may offer a viable therapeutic option for targeting this subpopulation.

IOMM‐Lee is a patient‐derived cell line that necessitates the use of an immunocompromised host to prevent graft rejection.^[^ ^40^ ^]^ This limits the ability to study the full immune landscape and responses to immunotherapies. Here, we have established a human meningioma model in mice with a reconstituted human immune system, demonstrating effective tumor reprogramming upon treatment with the nanoparticles. Tumor growth rates were significantly reduced, and survival was extended, suggesting that this model may offer valuable insights for future studies on meningioma immunotherapy, and indicating that this treatment paradigm warrants further exploration.

Results from the intracranial, orthotopic CT‐2A model are particularly promising, given the formidable challenge posed by the blood–brain barrier, which maintains the brain as an immune‐privileged site. In addition to improving median survival, we observed increased T‐cell infiltration and a complete resolution of tumors in 35% of treated mice. Furthermore, IHC results from the tumor demonstrate increased CD8 cell infiltration in various treatment arms of our experiment, especially when anti‐PD‐1 and 4‐1BBL/IL‐12 were used in combination. These results indicate that the combination therapy may effectively mobilize a localized immune response capable of sustaining tumor clearance. However, due to the complexity of human CNS tumors, with their heterogeneous cell populations, further investigation is required to ascertain how effective this treatment is at eradicating tumors from multiple locations. The implantation of patient‐derived xenografts in intracranial models will be critical to assess the true translational potential of this strategy and its ability to overcome the unique challenges posed by the CNS tumor microenvironment.

Our group has recently investigated the 4‐1BBL/IL‐12 tumor reprogramming strategy across several murine models, including Merkel cell carcinoma, colorectal carcinoma, breast cancer, and melanoma.^[^ ^20^, ^21^, ^41^ ^]^ The current study extends those findings to CNS tumors, which have a substantially different immune microenvironment compared to heterotopically implanted flank tumors, offering a potentially universal therapeutic platform applicable to various types of brain neoplasms. Ultimately, the data provide a foundation for further research into how nonviral nanoparticle‐mediated transient transfection can harness the adaptive immune system to combat lethal diseases like GBM and meningioma while minimizing off‐target effects. In the case of invasive glioma, it is often impossible to remove the infiltrating roots of the glioma during resection, thus leaving some malignant cells behind. Gliadel, the only Food and Drug Administration (FDA)‐approved local therapy for glioma, is specifically approved for initial surgery as well as the inevitable recurrence, as the operation during which the resection takes place provides an ideal opportunity for administration of local therapies.^[^ ^42^ ^]^ Similarly, this local adjuvant (4‐1BBL/IL‐12 PBAE nanoparticles) is beneficial to treat the tumor that cannot be removed or visualized because it infiltrates normal brain and can likewise be clinically relevant for eliminating residual brain tumors at a postsurgical resection site or in the case of unresectable tumors. Though we did not resect the majority of the tumor in this study, the 4‐1BBL/IL‐12 nanoparticles were still able to have a significant and beneficial effect on mouse survival. Focused ultrasound (FUS) has emerged as a potent technology for selectively disrupting the blood–brain barrier to increase the targeted delivery of chemotherapies and nanoparticles.^[^ ^43^, ^44^ ^]^ The potential for the present immune reprogramming strategy to be coupled with emerging technologies such as FUS to allow systemic delivery and targeted uptake warrants further exploration. Additional studies in humanized tumor models, as well as optimization of nanoparticle formulations, will be essential for realizing the full translational potential of this approach.

## 4 Experimental Section

4.1

4.1.1

##### Cell Lines

CT‐2A is a murine glioma cell line representing stem‐like cells in GBM, often conferring treatment resistance.^[^ ^22^ ^]^ CT‐2A cells (generously provided by Dr. Christopher Jackson, Johns Hopkins University) were cultured in DMEM (Sigma Aldrich D‐6429) supplemented with 1% Penicillin/Streptomycin (ThermoFisher) and 10% fetal bovine serum (Sigma Aldrich ES‐009‐B). IOMM‐Lee cells are a patient‐derived meningioma cell line. IOMM‐Lee cells (ATCC, CRL‐3370) were cultured in RPMI, 1% Penicillin/Streptomycin (ThermoFisher), and 10% fetal bovine serum (Sigma Aldrich ES‐009‐B).

##### Firefly Luciferase Tagging

CT‐2A cells were tagged with the Firefly Luciferase gene by transduction using a lentiviral vector with a puromycin resistance gene (BioScience, catalog number: 79 692‐P). Briefly, 150 000 CT‐2A cells were cultured with the lentiviral vectors at a multiplicity of infection (MOI) value of 10 and 8 mg mL^−1^ polybrene. After 24 h, the media was changed into puromycin‐containing media (10 mg mL^−1^, which was determined to be lethal for untransduced CT‐2A cells previously) for selection.

##### Piggybac Stable Expression of TDT+ in IOMM‐Lee Cells

IOMM‐Lee cells were transfected with the Piggybac transposase (System Biosciences, PB200A‐1) and transposon (Addgene plasmid # 120 870; http://n2t.net/addgene:120870; RRID:Addgene_120870) system containing luciferase and TDTomato as previously described.^[^ ^45^ ^]^ The nanoparticles used for the transfection were made in a 60 w/w, 600 ng DNA formulation in 20 μL. Cells were sorted twice for luciferase/TDTomato positive cells on the Sony SH800 fluorescence‐activated cell sorting (FACS) instrument to isolate a > 88% luciferase/TDTomato+ population.

##### Polymer Synthesis and Degradation Experiments

PBAEs are represented by three numbers separated by hyphens, the first referring to the backbone monomer, the second to the sidechain monomer, and the last to the end‐cap monomer that was used in their synthesis. Synthesis was conducted based on a previously published protocol.^[^ ^46^ ^]^ Briefly, a 1.1:1 molar ratio of acrylate backbone was mixed with an amine sidechain at 85 °C for 24 h before dissolving in anhydrous tetrahydrofuran at 200 mg mL^−1^. An end‐capping monomer was added to react at 25°C for 2 h, followed by precipitation in diethyl ether, washing, drying, and dissolution in anhydrous dimethyl sulfoxide (DMSO). Dissolved polymers were stored at −80°C with desiccant. 4‐5‐39 polymer degradation was measured after incubating the polymer in PBS for 0, 1, 2, 4, 8, and 24 h and analyzing molecular weight via gel permeation chromatography (1260 Infinity II with refractive index detector, Agilent).

##### In Vitro Nanoparticle Transfection

The transfection efficacy of the synthesized nanoparticles was established in IOMM‐Lee meningioma cells and CT‐2A glioma cells. 10 000 cells were seeded in each well of a 96‐well plate on Day 0 in 100 μL of media. On Day 1, polymers and DNA were diluted in 25 mM sodium acetate (NaAc), pH 5.2, to prepare 60 w/w, 600 ng of pEGFP‐N1 DNA in 20 μL of nanoparticles for each well. The DNA was mixed with polymer and allowed to complex for 10 min before being added dropwise to the cells. Two hours after transfection, the media in each well was replaced with 100 μL of fresh media. On Day 3, cells were washed with PBS, trypsinized, and then resuspended with FACS buffer (PBS, 2% FBS). Flow cytometry was performed on the cells using the Attune NxT Flow Cytometer (ThermoFisher; Waltham MA) to assess transfection efficiency.

For Lipofectamine comparison, Lipofectamine 2000 (ThermoFisher; Waltham MA) was tested against a PBAE formulation delivering 600 ng of EGFP‐N1 at 60 w/w to compare transfection efficiency and cell viability. The Lipofectamine conditions tested were 1:1 of DNA μg mass to μL Lipofectamine reagent volume. For the Lipofectamine nanoparticles, reagent and EGFP‐N1 plasmid were diluted in Opti‐MEM media (ThermoFisher; Waltham MA) according to the provided protocol. After 5 min of complexing, Lipofectamine nanoparticles were added to cells. PEI (linear, MW 25 000) (Polysciences, Inc; Warrington, PA) transfections were similarly conducted using previously published protocols for mammalian cell transfection.^[^ ^47^ ^]^ On the day following transfection, the 3‐(4,5‐dimethylthiazol‐2‐yl)‐5‐(3‐carboxymethoxyphenyl)‐2‐(4‐sulfophenyl)‐2 H‐tetrazolium (MTS) assay was conducted using CellTiter 96 AQueous One Solution Cell Proliferation Assay (Promega; Madison, WI) following the manufacturer's protocol. Statistical analysis was carried out using one‐way ANOVA with Dunnett's post‐test compared to the 4‐5‐39 condition in the IOMM‐Lee cells and compared to the 5‐3‐49 condition in the CT‐2A cells.

##### Nanoparticle Characterization

For the in vitro studies, twelve nanoparticles were formulated at 60 w/w (polymer to DNA mass ratio) with 600 ng pEGFP‐N1 plasmid (purchased from Clontech, amplified by Elim Biopharmaceuticals; Hayward, CA) in 20 μL. Nanoparticle sizes, polydispersity indices, and zeta potentials were measured using a ZetaSizer Pro (Malvern Panalytical; Malvern, UK) after dilution in 0.1X PBS. Encapsulation efficiency was measured using the Quant‐iT RiboGreen RNA Reagent (ThermoFisher; Waltham MA). For in vivo studies, nanoparticles for the CT‐2 A study, 4‐5‐39 (GFP and Signal 2/3, 15 w/w, 0.1 mg mL^−1^ DNA) and 5‐3‐49 (GFP and Signal 2/3, 15 w/w, 0.1 mg mL^−1^ DNA), and for the IOMM‐Lee study, 4‐5‐39 (GFP and Signal 2/3, 30 w/w, 0.2 mg mL^−1^ DNA) were also measured via DLS on the ZetaSizer Pro.

##### PBMC Leukopack extraction

PBMCs were isolated from blood from a deidentified donor from a Leukopak collected by the Hemapheresis and Transfusion Center (Johns Hopkins Hospital). Briefly, blood was mixed with complete RPMI media and Ficoll‐Hypaque in conical 50 mL tubes. After centrifuging for 30 min at 400 × g, the mononuclear cell layer was extracted and centrifuged for an additional 10 min. The supernatant was then removed, and the cells were counted. Cells were then resuspended in freezing medium (90% FBS + 10% DMSO) and dispensed into cryovials containing 10^8^ cells per 1 mL aliquot.

##### Astrocyte/Meningioma Coculture

A total of 10^4^ NHA Human Astrocytes (Lonza; Basel, Switzerland) and IOMM‐Lee cells were seeded on Day 0, at 1:1, 10:1, or 5:1 ratios of IOMM‐Lee to human astrocytes. Cells were seeded and transfected on Day 1 with 4‐5‐39 nanoparticles at 60 w/w, 600 ng DNA. On Day 3, cells were imaged via fluorescent microscopy and analyzed by flow cytometry. Under fluorescence microscopy, IOMM‐Lee fluoresced red due to stable expression of TDTomato. Healthy astrocytes expressing GFP appeared green, and IOMM‐Lee cells with stable expression of TDTomato and transient expression of GFP appeared yellow. Statistical analysis was carried out using two‐way ANOVA with Sidak's post‐test.

##### Immunological Signals Expression (Signal 2s, IL‐12]

To assess expression of various Signal 2 molecules, IOMM‐Lee cells were seeded at 10 000 cells per well and transfected with 4‐5‐39 nanoparticles carrying pUNO human 4‐1BBL (InvivoGen; San Diego, CA), pUNO mouse 4‐1BBL (InvivoGen; San Diego, CA), pUNO CD80 (InvivoGen; San Diego, CA), and pUNO CD86 (InvivoGen; San Diego, CA). 48 h after transfection, cells were stained with antibodies against Signal 2 molecules (**Table** **3**) and measured via flow cytometry. To measure IL‐12 expression, cells were transfected with 4‐5‐39 nanoparticles containing either 100% EGFP‐N1, 100% 4‐1BBL, 100% IL‐12, 50% eGFP, and 50% 4‐1BBL, 50% eGFP and 50% IL‐12 (InvivoGen; San Diego), or 50% 4‐1BBL and 50% IL‐12, and 48 h after transfection, the cell supernatant was harvested for an IL‐12 ELISA assay (ELISA MAX Deluxe Set Human IL‐12, Biolegend; San Diego, CA) following the manufacturer's protocol. Statistical analysis was carried out using one‐way ANOVA with Dunnett's post‐test comparing all groups to GFP the condition.

**Table 3:** Antibodies used for flow cytometry.

Fluorophore	Target	Clone	Manufacturer	Catalog no.	Dilution
PE	Anti‐human 4‐1BB Ligand	5F4	Biolegend	311 503	80
Brilliant Violet 421	Anti‐mouse CD80	16‐10A1	Biolegend	104 725	20
PE	Anti‐mouse 4‐1BB Ligand (CD137L)	TKS‐1	Biolegend	107 105	80
Brilliant Violet 605	Anti‐mouse CD86	GL‐1	Biolegend	105 037	80
PE	Anti‐human CD3	SK7	Biolegend	981 004	100
Brilliant Violet 785	Anti‐human CD45	HI30	Biolegend	304 048	20
PerCP/Cyanine5.5	Anti‐human HLA‐DR, DP, DQ	Tü39	Biolegend	361 710	20

##### Tumor/PBMC or Tumor/Splenocyte Coculture

Activation of the immune system in response to Signal 2 and Signal 3 tumor reprogramming was evaluated using coculture with leukocytes isolated from the spleens of C57BL/6 mice for murine CT‐2A cells or PBMCs isolated from a human donor for human IOMM‐Lee cells, followed by an ELISA to assess IFN‐gamma production. All statistical analysis was carried out using one‐way ANOVA with Dunnett's post‐test comparing all groups to the GFP condition. On Day 0, 10 000 CT‐2A or IOMM‐Lee cells were cultured per well in a 96‐well plate.

*IOMM‐Lee*: On Day 1, 4‐5‐39 nanoparticles at 60 w/w containing either 100% EGFP‐N1, 100% 4‐1BBL, 100% IL‐12, 50% EGFP and 50% 4‐1BBL, 50% EGFP and 50% IL‐12, or 50% 4‐1BBL and 50% IL‐12 were added to tumor cells. On Day 2, either 20 K or 50 K PBMCs were added to the cells. 48 h after transfection, the supernatant was collected for an IFNγ ELISA (LEGEND MAX Human IFN‐γ ELISA Kit; Biolegend; San Diego, CA).

##### CT‐2A

On Day 1, 4‐5‐39 or 5‐3‐49 nanoparticles were formulated with either GFP (control), Signal 2 (4‐1BBL), Signal 3 (IL‐12), or Signal 2 and 3 at 90 w/w. Tumor cells were transfected with nanoparticles containing 100% EGFP, 100% 4‐1BBL, 100% IL‐12, 50% eGFP, and 50% 4‐1BBL, 50% EGFP, and 50% IL‐12, or 50% 4‐1BBL and 50% IL‐12. On Day 2, 200 K splenocytes were added into each well. 72 h after transfection, the cell supernatant was harvested for an IFN‐gamma ELISA (ELISA IFN‐Gamma, Biolegend; San Diego, CA).

##### Ex Vivo Reprogramming

IOMM‐Lee cells were seeded at 10 000 cells per well. 24 h after seeding, they were transfected with 4‐5‐39 nanoparticles containing either 100% EGFP‐N1, 100% 4‐1BBL, 100% IL‐12, 50% eGFP, and 50% 4‐1BBL, 50% eGFP and 50% IL‐12, or 50% 4‐1BBL and 50 % IL‐12. 4‐1BBL and IL‐12 plasmids were purchased from Invivogen (San Diego, CA). 48 h after seeding, PBMCs isolated from a Leukopak were added to the wells at either 20 K or 50 K PBMCs per well. Each condition was performed in quadruplicates. 72 h after seeding, cells were washed with 100 μL PBS, trypsinized, stained for antibodies against CD3, CD45, and MHC II, and measured on the Attune NxT Flow Cytometer. Statistical analysis was carried out using one‐way ANOVA with Dunnett's post‐test comparing all groups to the GFP condition.

##### Surgical Protocol for Intracranial Procedures

All animal procedures were performed in compliance with approved protocols (MO24M284, MO23E357, and MO24M346) by the Johns Hopkins University Animal Care and Use Committee (ACUC). The following protocol was followed for all in vivo studies involving intracranial procedures (orthotopic tumor implantation, CED of nanoparticles). Mice were anesthetized via intraperitoneal injection of 200 mL of a preprepared mixture of Xylazine (2.5 mg kg^−1^), Ketamine (25 mg kg^−1^), and ethanol (14.25%) in 0.9% sterile saline. For analgesia, the mice received a subcutaneous injection of 0.5–1.0 mg kg^−1^ Buprenorphine sustained release (SR). Upon confirmation of proper anesthesia, the mice were prepared for surgery. This included shaving the scalp (in C57BL/6 mice), applying 70% alcohol and Prepodyne to the surgical site, and creating a midline incision. Subsequently, a burr hole with a 2 mm diameter was drilled with its center located 2 mm posterior and 2 mm lateral (left‐side) to Bregma, while preserving the dural integrity. Next, durotomy was performed under the guidance of a surgical microscope (Zeiss OPMI CS NC‐2 surgical microscope). The animal was then placed on a stereotactic frame for either cell injection or CED delivery (described later) of nanoparticles. Finally, after the procedure was completed, the wound was closed using a sterile autoclip, and mice were monitored postoperatively until they emerged from anesthesia and were able to ambulate.

##### In Vivo Orthotopic Transfection for IOMM‐Lee

10 athymic nude mice (Nu/J 002019; Jackson Laboratories, Bar Harbor, ME) were intracranially injected with 1 million (3 mice), 500 K (3 mice) or 250 K (4 mice) IOMM‐Lee cells to establish a tumor volume standard curve. Their weights were monitored for 13 days, and the tumors were monitored via an IVIS (Revvity Lumina III). For the orthotopic transfection study, 500 K IOMM‐Lee cells were implanted into the brains of 40 mice. GFP nanoparticles were made as described above using EGFP‐N1 plasmid DNA and 4‐5‐39 polymer, diluted in 30 mM NaAc at pH 5, then coformulated with sucrose as a cryoprotectant at a final concentration of 90 mg mL^−1^ and frozen at −80°C until use. 6 days following tumor implantation, 8 μL of nanoparticles were injected into the mouse brains via CED. 24 h after nanoparticle delivery, the mice were euthanized, the brains were harvested, and a hemispherectomy was performed to isolate the lobe with the tumor. GFP concentration was measured using the provided protocol from a GFP ELISA Kit (Ab171581, Abcam; Cambridge, UK).

##### In Vivo Heterotopic IOMM‐Lee survival

16 hu‐PBMC‐NSG MHC I/II Double Knockout (742 516 Jackson Labs; Bar Harbor, ME) mice were implanted with 1E6 IOMM‐Lee cells in the flank. This model was selected because it enables engraftment of the human IOMM‐Lee meningioma cells while also allowing for human T‐cell function. The MHC I/II double knockout makes this mouse strain ideal for immuno‐oncology studies because it reduces xenograft‐versus‐host disease (GvHD), allowing for longer studies. Mice were shaved and their flanks prepared with 70% alcohol before subcutaneous implantation. Tumor width and length were measured every two days to provide an area estimate of the tumor size, and when tumors were ≈25 mm^2^ in area, mice were placed into two groups so that the average size in each group was similar. 4‐5‐39 nanoparticles at 30 w/w, 0.2 mg mL^−1^ containing either 100% GFP or 50% 4‐1BBL and 50% IL‐12 were injected intratumorally on Days 9, 11, 13, 23, 25, and 27. Tumor sizes were measured every two days from Day 9, and mice were euthanized when tumors reached 200 mm^2^. Areas were converted to volume by multiplying the longer dimension between length and width by the square of the shorter dimension and dividing by two. Statistical analysis was carried out using Log‐rank (Mantel‐Cox) test.

##### In Vivo Transfection for CT‐2A

C57BL/6 mice for all in vivo glioma experiments were purchased from Charles Rivers Laboratories (Rockville, MD). *In vivo*, transfection efficacy was evaluated in both flank and orthotopic murine models using the CT‐2A murine glioma cell line. For the flank CT‐2 A model, on Day 0, 10 C57BL/6 mice were inoculated with 5 million CT‐2A cells suspended in 50% PBS and 50% Matrigel (Corning Inc., Corning, NY). On Day 7, mice were injected intratumorally with nanoparticles (5‐3‐49 and 5‐3‐27) complexed with the firefly luciferase gene to evaluate luciferase expression in vivo, with 10 μg DNA and 50 μL volume injected per mouse. On Day 8, mice were given 200 μL of 15 mg mL^−1^ D‐Luciferin (GoldBio) per mouse subcutaneously for bioluminescent imaging using IVIS (Revvity Lumina III). To evaluate the level of transfection into intracranial tumors, 32 mice were inoculated with 125 000 CT‐2A cells. 7 days after inoculation, nanoparticles complexed with EGFP‐N1 plasmid were injected intratumorally. 8 μL of nanoparticle solution were injected over 5 min using CED. 24 h after transfection, mice were euthanized, and the brain was excised. A hemispherectomy was performed to isolate the hemisphere inoculated with the tumor. The tumor was extracted, homogenized, and lysed for GFP ELISA to measure total GFP in the ipsilateral hemisphere of the brain.

##### IVIS Imaging

Mice were anesthetized using Fluriso isoflurane, and then injected subcutaneously with 3.75 to 4 mg luciferin 10 min before images were taken via IVIS.

##### In Vivo Heterotopic CT‐2A Survival

64 mice were inoculated in the left flank with 5E6 CT‐2A cells suspended in 50% PBS and 50% Matrigel (Corning Inc., Corning, NY). 7 days after inoculation, the mice were randomized into the following treatment groups (*n* = 16): 1) intratumoral 4‐1BBL/IL‐12 nanoparticles with systemically delivered anti‐PD‐1, 2) intratumoral 4‐1BBL/IL‐12 nanoparticles only, 3) intratumoral GFP nanoparticles with systemically delivered anti‐PD‐1, or 4) intratumoral GFP nanoparticles only. At 7, 9, and 11 days postinoculation, nanoparticles were intratumorally injected coupled with PD‐1 blockade (5 mg kg^−1^) (BioXcell, cat. BE0146), which was administered intraperitoneally for the appropriate groups. Mice were monitored daily for tumor width and length, and euthanized when they met the Johns Hopkins University Animal Care and Use Committee criteria of length greater than 20 mm or area greater than 200 mm^2^, or for moribund states. The groups were blinded to the team conducting daily measurements and evaluating mice for euthanasia.

##### In Vivo Orthotopic CT‐2A Survival

For the intracranial study, 160 mice were intracranially inoculated with 75 000 CT‐2A cells. Five days after inoculation, mice received an intratumoral injection of 7 μL of nanoparticle solution at 2 μL min^−1^ using a CED device with a 50 μL Hamilton Neuros Syringe (Hamilton, Reno, NV), after which mice were kept on the stereotactic frame for 4 min before the Hamilton syringe was withdrawn. Mice were randomized into eight groups (*n* = 20) as follows: 1) 5‐3‐49 nanoparticles loaded with GFP DNA (control), 2) 5‐3‐49 nanoparticles loaded with GFP DNA + systemically delivered anti‐PD‐1, 3) 5‐3‐49 nanoparticles loaded with 4‐1BBL/IL‐12 DNA, 4) 5‐3‐49 nanoparticles loaded with 4‐1BBL/IL‐12 DNA + anti‐PD‐1, 5) 4‐5‐39 nanoparticles loaded with GFP DNA, 6) 4‐5‐39 nanoparticles loaded with GFP DNA + systemically delivered anti‐PD‐1, 7) 4‐5‐39 nanoparticles loaded with 4‐1BBL/IL‐12 DNA, and 8) 4‐5‐39 nanoparticles loaded with 4‐1BBL/IL‐12 DNA + systemically delivered anti‐PD‐1. On Days 5, 8, and 12 postinoculation, mice received treatment as indicated. Anti‐PD‐1 therapy (5 mg kg^−1^) (BioXcell, cat. BE0146) was administered through intraperitoneal injection. Mice received buprenorphine injection before surgery for pain relief and were provided gel cups for recovery between surgeries. They were monitored daily for the development of any deleterious signs requiring euthanasia as per the JHU ACUC guidelines. The team responsible for monitoring mice for euthanasia was blinded to the groups of the experiment. 72 h after the final treatment, three mice from each group were randomly selected and euthanized and their brains were harvested and stored in 10% formalin. The brains were subsequently embedded in paraffin and sectioned for IHC and H&E staining. Statistical analysis for in vivo survival studies was performed using Kaplan–Meier survival plots, and survival curves were compared using the log‐rank (Mantel‐Cox) test.

The study was replicated in 80 mice that were intracranially inoculated with 125 000 luciferase‐tagged CT‐2A cells. Mice were randomized into four groups (*n* = 16–17). On Days 7, 11, and 14 postinoculation, mice received 1) 5‐3‐49 nanoparticles loaded with GFP DNA (control), 2) 5‐3‐49 nanoparticles loaded with GFP DNA + systemically delivered anti‐PD‐1, 3) 5‐3‐49 nanoparticles loaded with 4‐1BBL/IL‐12 DNA, or 4) 5‐3‐49 nanoparticles loaded with 4‐1BBL/IL‐12 DNA + anti‐PD‐1. On each treatment day, the mice received an intratumoral injection of 8 μL of treatment using a CED device with a Hamilton Neuros Syringe at a rate of 2 μL min^−1^ (Hamilton, Reno, NV). Mice received buprenorphine injection before surgery for pain relief and were provided gel cups for recovery between surgeries. Mice were monitored daily for any deleterious signs requiring euthanasia.

##### Tumor Volume Measurement

In the orthotopic efficacy experiment with CT‐2A tumors, three mice from each group were randomly selected and euthanized 72 h after the final treatment. Their brains were harvested, fixed in 10% formalin, and embedded in paraffin. Subsequently, twelve sections spanning the tumor were prepared, with nine of those sections stained using H&E staining (performed by the Johns Hopkins Reference Histology Core). Each section was cut at 200 μm intervals. The area of the tumor in each slice was calculated using Zeiss AxioPro, and the volume was estimated by multiplying the area of each slice by the distance between slices. For any unstained slides, the area was estimated by averaging the areas of the adjacent stained sections.

##### Immunohistochemistry Staining

Immunohistochemical staining was performed at the Johns Hopkins University Oncology Tissue Services Core. Following dewaxing and rehydration of the paraffinized sections, antigen retrieval was done using Ventana Ultra CC1 buffer (Roche Diagnostics) at 96 °C for 64 min. Next, the primary rabbit anti‐mouse FoxP3 antibody (Cell Signalling Technology) was added and incubated at 36°C for 60 min. Subsequently, a secondary anti‐rabbit HQ followed by the anti‐HQ horseradish peroxidase (HRP) were added (Roche Diagnostics) followed by Chromomap DAB IHC detection kit (Roche Diagnostics). Following FOXP3 detection, primary and secondary antibodies from the first round of staining were stripped on board using Ventana Ultra CC1 buffer at 95°C for 12 min. The primary rabbit anti‐mouse CD4 antibody (Abcam) was added and incubated at 36°C for 60 min. Then the secondary anti‐rabbit HQ followed by the anti‐HQ HRP were added (Roche Diagnostics) followed by Discovery Purple IHC detection kit (Roche Diagnostics). Following CD4 detection, primary and secondary antibodies from the second round of staining were stripped on board using Ventana Ultra CC1 buffer at 95°C for 12 min. Finally, the primary rat anti‐mouse CD8 (Invitrogen) was added and incubated at 36°C for 60 min, followed by rabbit anti‐rat linker antibody (Vector Labs) at 36°C for 32 min. The linker rabbit antibody was detected using an anti‐rabbit HQ followed by anti‐HQ HRP (Roche Diagnostics) followed by the application of Discovery Teal Detection kit (Roche Diagnostics). Finally counterstaining with Mayer's hematoxylin, dehydration, and mounting were performed. All antibody information is listed in **Table** **4**.

**Table 4:** Antibodies used for IHC.

Antibody	Manufacturer	Catalog no.	Dilution
Rabbit anti‐mouse FoxP3	Cell signaling technology	12653S	50
Rabbit anti‐mouse CD4	Abcam	ab183685	1000
Rat anti‐mouse CD8	Invitrogen	14‐0195‐82	125
Anti‐rabbit HQ	Roche diagnostics	07 017 812 001	–
Anti‐HQ HRP	Roche diagnostics	07 017 936 001	–
Rabbit anti‐rat IgG linker	Vector labs	AI4001	500

## Supporting Information

Supporting Information is available from the Wiley Online Library or from the author.

## Conflict of Interest

H.B. is a paid consultant to Insightec and chairman of the company's Medical Advisory Board. Insightec is developing focused ultrasound treatments for brain tumors. This arrangement has been reviewed and approved by the Johns Hopkins University in accordance with its conflict‐of‐interest policies. H.B. receives research funding from NIH, Johns Hopkins University, Khatib Foundation, NICO Myriad Corporation, and philanthropy. H.B. is a consultant for Candel Therapeutics, Inc., Catalio Nexus Fund II, LLC, LikeMinds, Inc*, Galen Robotics, Inc.* CraniUS*, and Nurami Medical* (*includes equity or options). B.T. has research funding from NIH and is a co‐owner for Accelerating Combination Therapies*. Ashvattha Therapeutics Inc. has also licensed one B.T.'s patents, and she is a stockholder in Peabody Pharmaceuticals (*includes equity or options). Johns Hopkins filed patents related to the technology discussed in the manuscript with J.J.G. as a co‐inventor. J.J.G. is also a board member, CSO, and co‐founder of Cove Therapeutics, a manager, CTO, and co‐founder of Dome Therapeutics, and a manager and co‐founder of OncoSwitch Therapeutics. S.Y.T. is a manager and co‐founder of OncoSwitch Therapeutics. Any potential conflicts of interest are managed by the Johns Hopkins University Committee on Outside Interests. There are no competing interests from the other authors.

## Supporting information

Supplementary Material

## Data Availability

Additional data and materials used in this study are available upon request to JJG following JHU policies.
