# Supplementary material for: DNA‐Loaded Nanoparticles Reprogram the Tumor Immune Microenvironment to Treat Brain Tumors
Source: Small Sci. 2026 Jan 5;6(1):e202500475. doi: 10.1002/smsc.202500475 (PMC12767759; doi:10.1002/smsc.202500475)
Supplement: Supplementary file 1 — Supplementary Material [file SMSC-6-e202500475-s001.pdf]

Supporting Information

Title: DNA-loaded Nanoparticles Reprogram the Tumor Immune Microenvironment to Treat Brain Tumors

Author(s), and Corresponding Author(s):* Joanna Yang, Divyaansh Raj, Hasan Slika, Aanya Shahani, Leonardo Cheng, Manav Jain, Ethan Idnani, Kathryn Luly, FNU Ruchika, Caitlin Kraft, Charles Eberhart, Henry Brem, Betty Tyler, Jordan J. Green, Stephany Y. Tzeng

Supporting Information

Figure S1. Geometric mean GFP fluorescence intensity and cell counts after transfection with PBAE nanoparticle library.

Figure S2: PBAE nanoparticle transfection geometric mean GFP fluorescence intensity and viability (MTS) compared to PEI and Lipofectamine 2000.

Figure S3. Degradation of 4-5-39 at pH 7.

Figure S4: IOMM-Lee Meningioma cells co-cultured with healthy human astrocyte (HA) cells demonstrate certain PBAE nanoparticle gene delivery tropisms.

Figure S5. Co-culture of IOMM-Lee cells with healthy human astrocyte (HA) cells at 1:1 and 1:5 ratio to assess PBAE tropism.

Figure S6. Ex vivo study in IOMM-Lee cells with 50K PBMCs.

Figure. S7: Flank and orthotopic transfection of IOMM-Lee and CT-2A tumors with PBAE nanoparticles.

Figure S8. Pilot studies with IOMM-Lee cells in humanized mice to assess tumor growth.

Figure S9. Characterization of *in vivo* nanoparticles.

Figure S10. Meningioma tumor tissues IHC at 5X.

Figure S11. Kaplan Meier curve for the in vivo study with firefly luciferase-tagged CT-2A tumors and tumor volume comparisons for the 5-3-49 nanoparticle cohort and 4-5-39 nanoparticle cohort in CT-2A tumors.

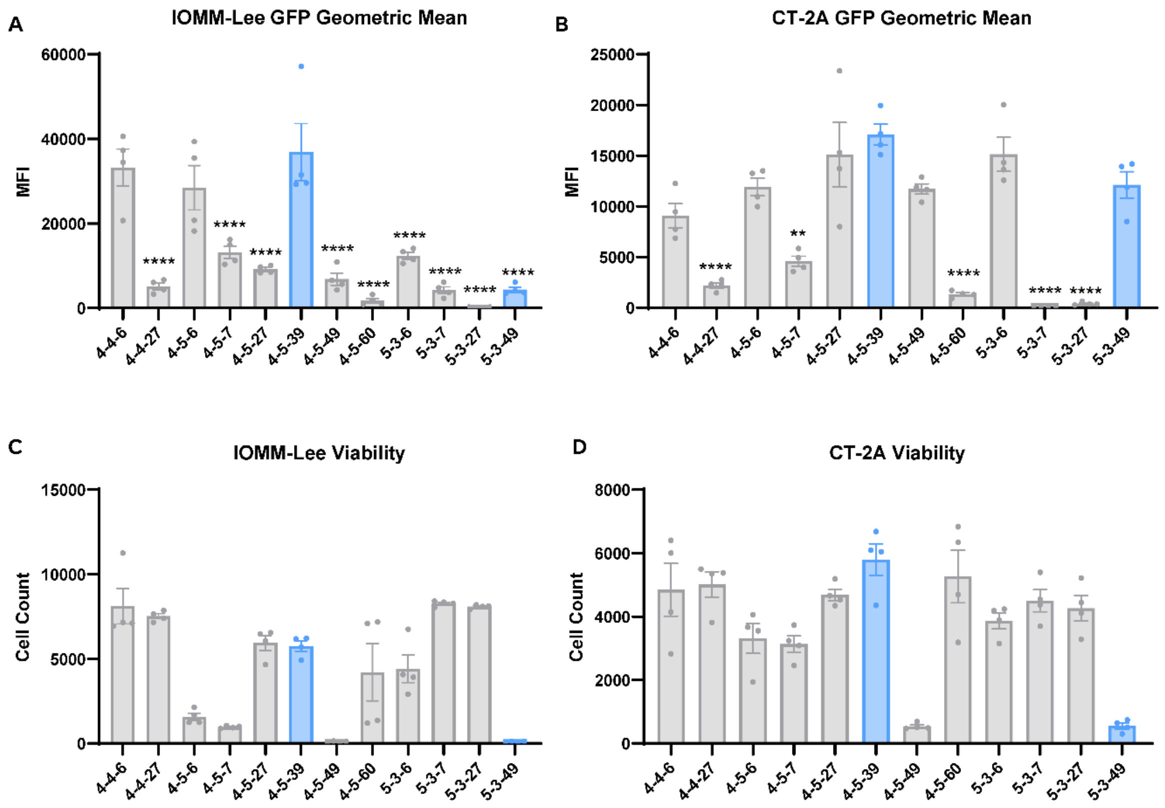

Figure S1: Geometric mean GFP fluorescence intensity and cell counts after transfection with PBAE nanoparticle library. (**A**) Geometric mean GFP fluorescence intensity of IOMM-Lee cells after transfection (one-way ANOVA, Dunnett's test, compared to 4-5-39). (**B**) Geometric mean GFP fluorescence intensity of CT-2A cells after transfection (one-way ANOVA, Dunnett's test, compared to 5-3-49). (**C** to **D**) Cell counts of IOMM-Lee and CT-2A cells two days after transfection as an indicator of viability. Significance is represented by *P ≤ 0.05, **P ≤ 0.01, ***P ≤ 0.001, and ****P ≤ 0.0001. Each data bar represents mean ± SEM with four technical replicates.

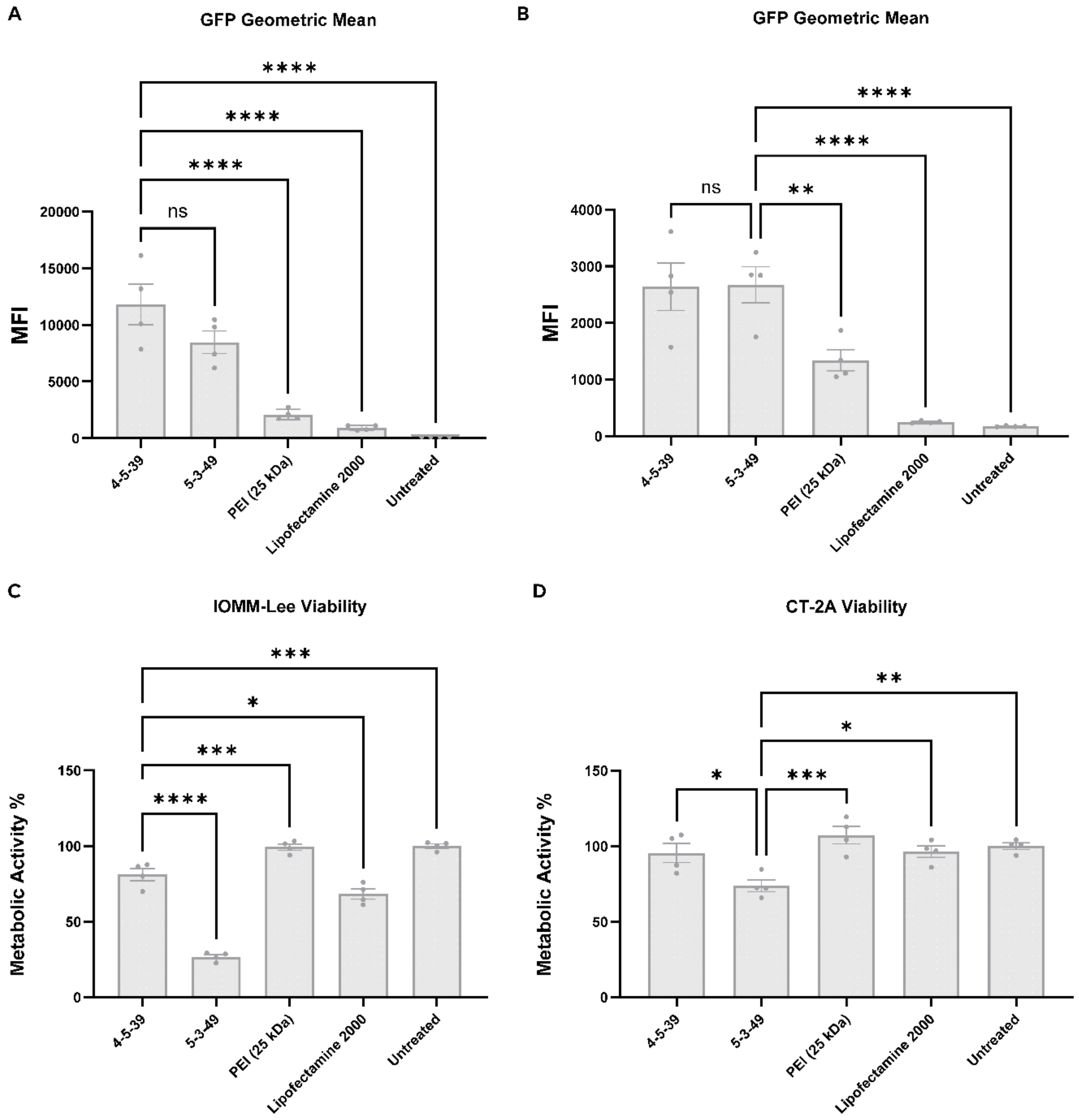

Figure S2: PBAE nanoparticle transfection geometric mean GFP fluorescence intensity and viability (MTS) compared to PEI and Lipofectamine 2000. (**A**) Geometric mean GFP fluorescence intensity of IOMM-Lee transfections (one-way ANOVA, Dunnett's test, compared to 4-5-39). (**B**) Geometric means GFP fluorescence intensity of CT-2A transfections (one-way ANOVA, Dunnett's test, compared to 5-3-49). (**C**) Cell metabolic activity of IOMM-Lee cells after treatment with PBAE nanoparticles or PEI and Lipofectamine 2000 measured via MTS

assay, normalized to untreated control cells (one-way ANOVA, Dunnett's test, compared to 4-5-39). (**D**) Cell metabolic activity of CT-2A cells after treatment with PBAE nanoparticles or PEI and Lipofectamine 2000 measured via MTS assay, normalized to untreated control cells (one-way ANOVA, Dunnett's test, compared to 5-3-49). Significance is represented by *$P \leq 0.05$, **$P \leq 0.01$, ***$P \leq 0.001$, and ****$P \leq 0.0001$. Each data bar represents mean ± SEM with four technical replicates.

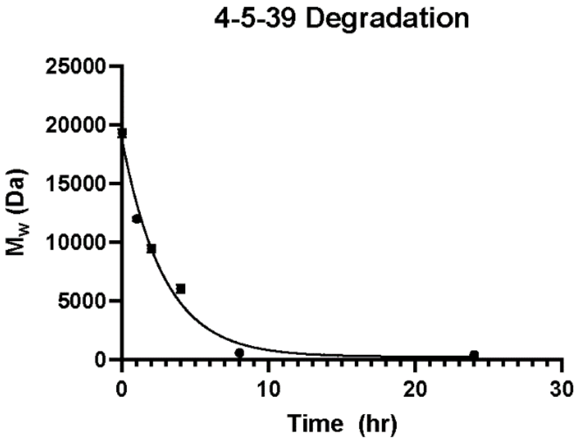

Figure S3. Degradation of 4-5-39 at pH 7. The 4-5-39 PBAE was incubated in pH 7 PBS for 0, 1, 2, 4, 8, and 24 hours to study degradation kinetics. At pH 7, the half-life of the 4-5-39 PBAE was 2 hours (non-linear regression, exponential one-phase decay).

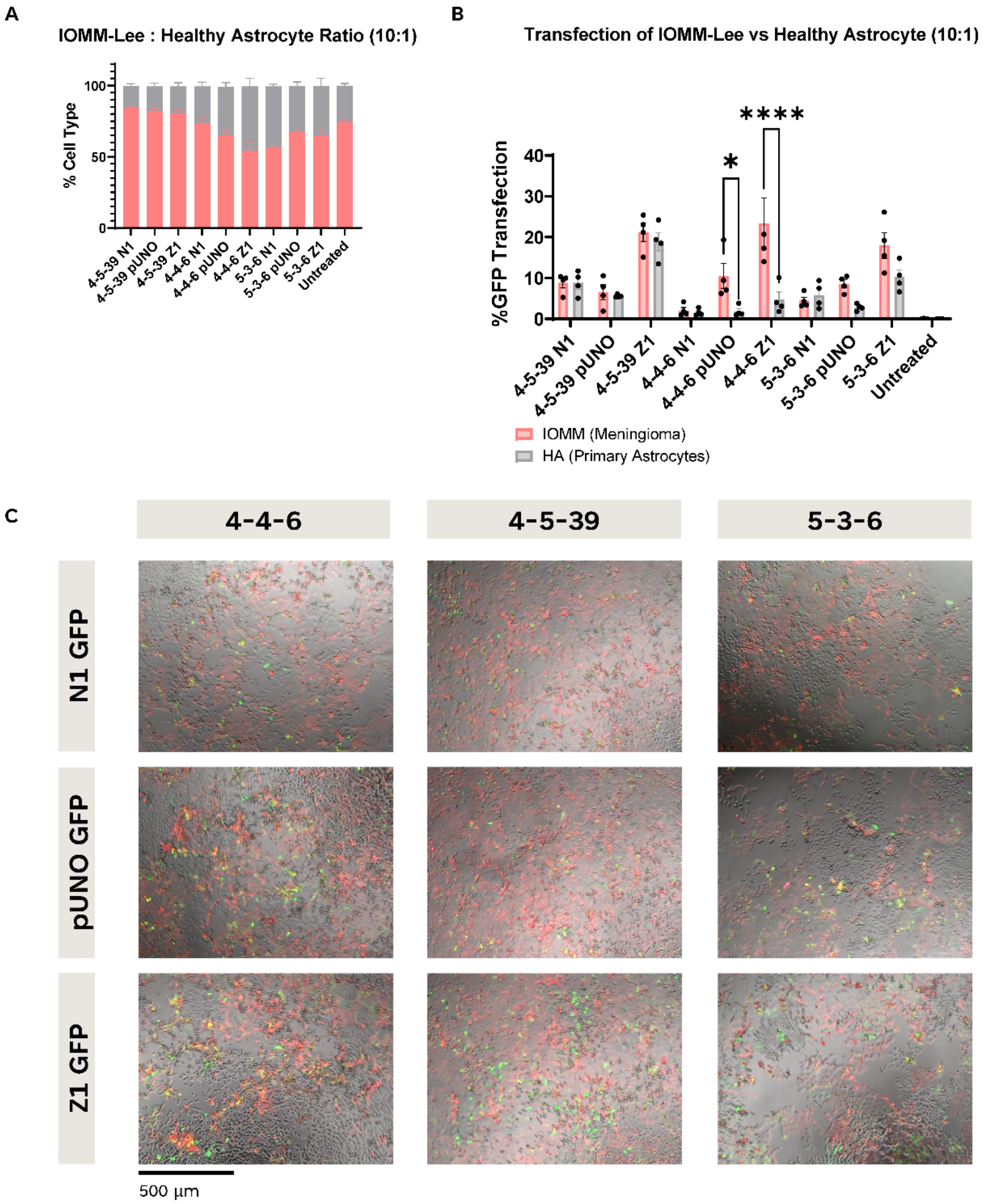

Figure S4. IOMM-Lee Meningioma cells co-cultured with healthy human astrocyte (HA) cells demonstrate certain PBAE nanoparticle gene delivery tropisms. (**A**) Cells were seeded at a 10:1 ratio of IOMM-Lee cells to healthy human astrocytes. Two days after transfection, the ratio of IOMM-Lee cells to HA cells are shown. (**B**) Some PBAE nanoparticle formulations

preferentially transfect IOMM-Lee meningioma cells over healthy human astrocyte cells when cultured together, across three different GFP reporter plasmids. In the 4-4-6 nanoparticles containing pUNO plasmids or Z1 plasmids, the PBAE nanoparticles transfected IOMM-Lee cells significantly higher than healthy human astrocytes (two-way ANOVA, Sidak's post-test). Significance is represented by $*P \leq 0.05$ and $****P \leq 0.0001$. (**C**) Fluorescence microscopy of the IOMM-Lee cells (TDTomato+) and GFP transfected cells are shown. Scale bars, 100 µm. Areas colored yellow indicate transfection of GFP into TDTomato+ IOMM-Lee cells. Each data bar represents mean ± SEM with four technical replicates. Scale bar represents 500 µm.

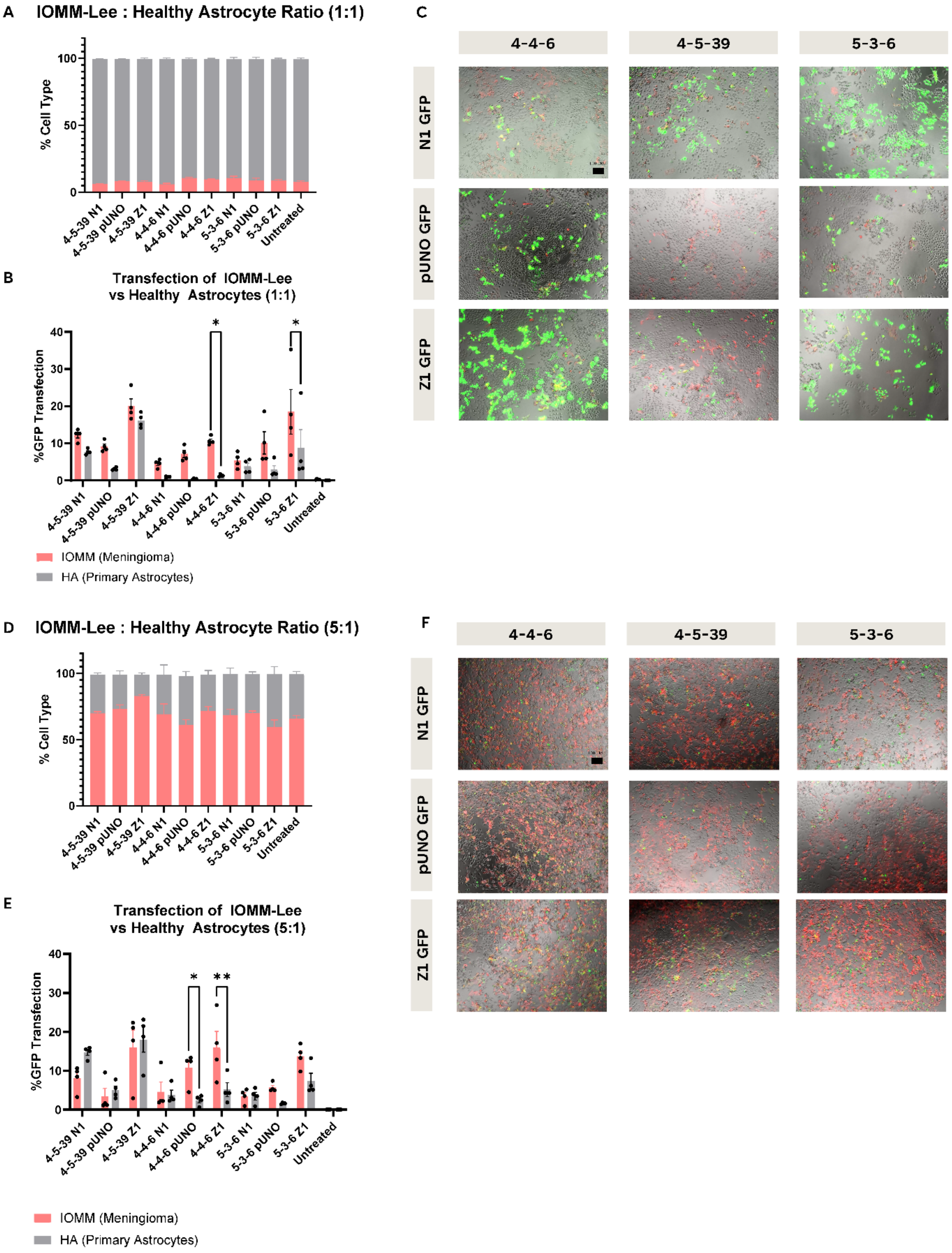
A
IOMM-Lee : Healthy Astrocyte Ratio (1:1)
% Cell Type
100
50
0
4-5-39 N1
4-5-39 pUNO
4-5-39 Z1
4-4-6 N1
4-4-6 pUNO
4-4-6 Z1
5-3-6 N1
5-3-6 pUNO
5-3-6 Z1
Untreated
B
Transfection of IOMM-Lee
vs Healthy Astrocytes (1:1)
%GFP Transfection
40
30
20
10
0
*
*
IOMM (Meningioma)
HA (Primary Astrocytes)
C
4-4-6
4-5-39
5-3-6
N1 GFP
pUNO GFP
Z1 GFP
D
IOMM-Lee : Healthy Astrocyte Ratio (5:1)
% Cell Type
E
Transfection of IOMM-Lee
vs Healthy Astrocytes (5:1)
%GFP Transfection
*
**
IOMM (Meningioma)
HA (Primary Astrocytes)
F
4-4-6
4-5-39
5-3-6
N1 GFP
pUNO GFP
Z1 GFP

Figure S5: Co-culture of IOMM-Lee cells with healthy human astrocyte (HA) cells at 1:1 and 1:5 ratio to assess PBAE tropism. (**A** to **C**) IOMM Lee cells were cultured at a 1:1 ratio with healthy human astrocytes. Fluorescence microscopy of the IOMM-Lee cells (Tdtomato+) and GFP transfected cells are shown. Areas colored yellow indicate transfection of GFP into Tdtomato+ IOMM-Lee cells. (**D** to **F**) IOMM Lee cells were cultured at 5:1 ratio with healthy human astrocytes. Fluorescence microscopy of the IOMM-Lee cells (Tdtomato+) and GFP transfected cells are shown. Areas colored yellow indicate transfection of GFP into Tdtomato+ IOMM-Lee cells. Scale bar represents 500 μm.

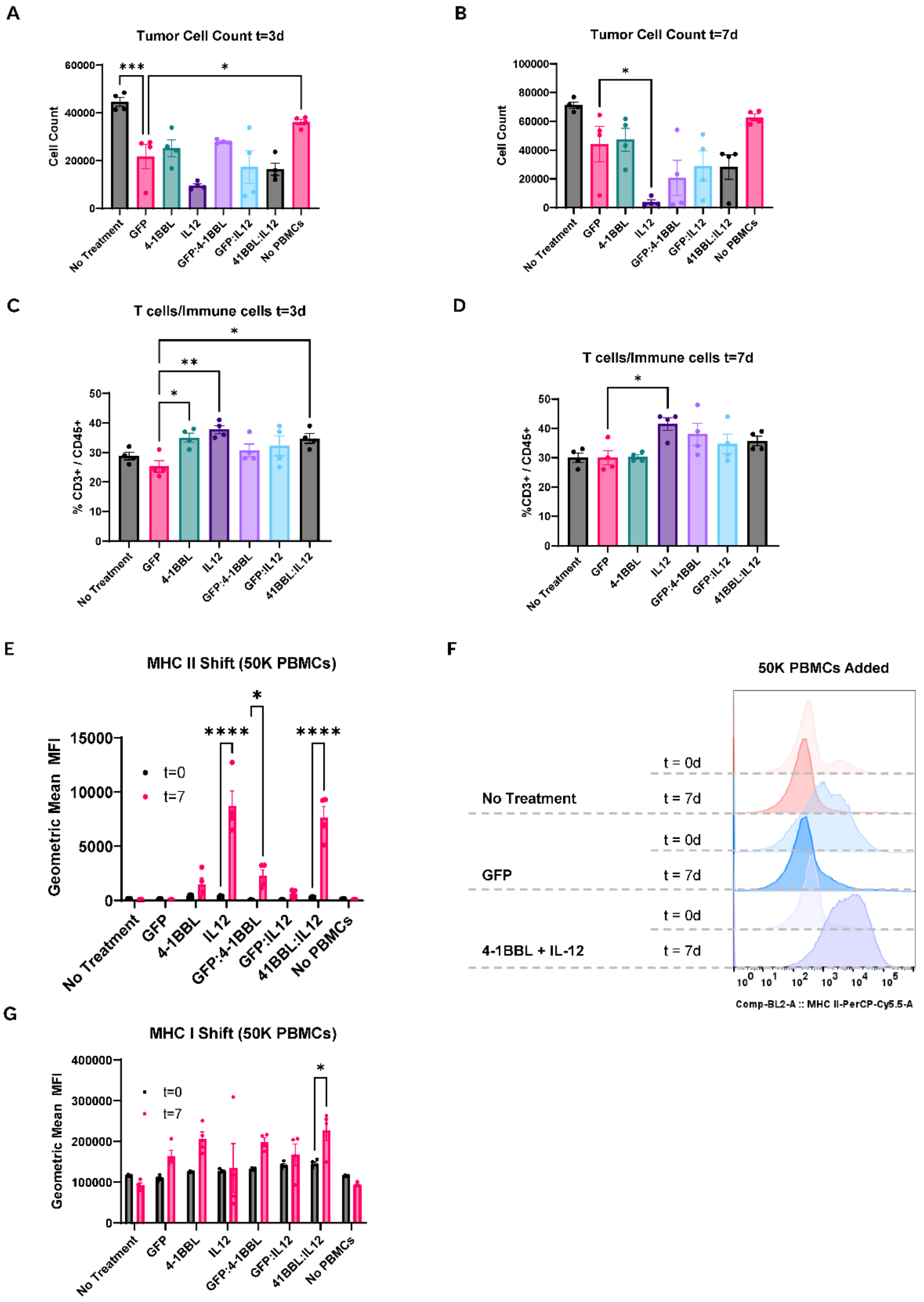

Figure S6: Ex vivo study in IOMM-Lee cells with 50K PBMCs. (**A** to **B**) Tumor cells were counted after 3 and 7 days. Although tumor cell count was lower after 7 days, there was no significant difference compared to treatment with the GFP nanoparticle. (**C** to **D**) Ratio of T-cells

to immune cells 3 days after PBMC addition and 7 days after is also depicted. (**E** to **F**) In treatment groups with IL12 or 4-1BBL/IL-12, there is a significant shift in MHC II expression after 7 days. (**G**) A significant shift in MHC I is also observed in groups receiving 4-1BBL/IL-12. Significance is represented by *P ≤ 0.05, **P ≤ 0.01, ***P ≤ 0.001, and ****P ≤ 0.0001. Each data bar includes means ± SEM with four technical replicates.

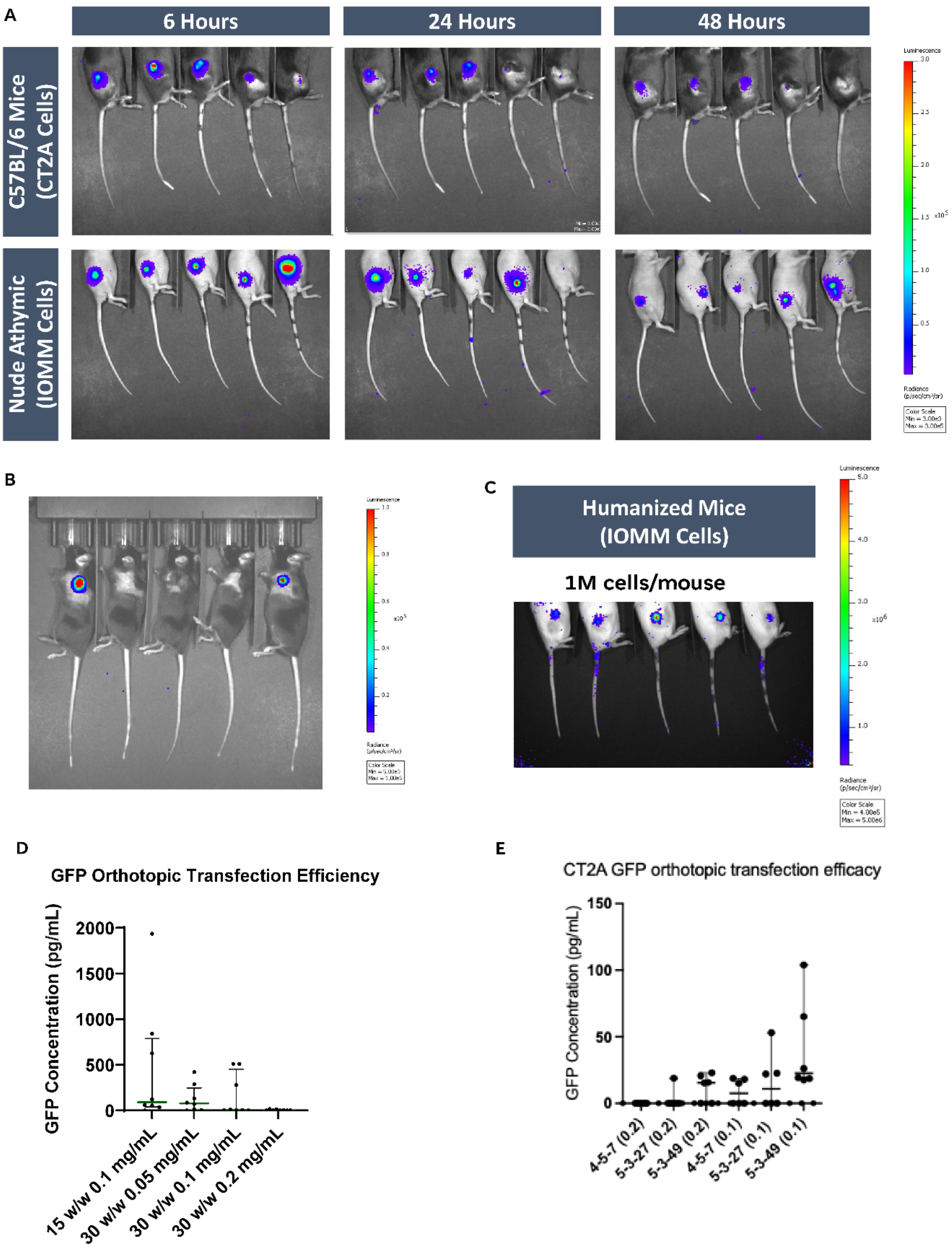
A
6 Hours
24 Hours
48 Hours
C57BL/6 Mice (CT2A Cells)
Nude Athymic (IOMM Cells)
B
C
Humanized Mice (IOMM Cells)
1M cells/mouse
D
GFP Orthotopic Transfection Efficiency
GFP Concentration (pg/mL)
2000
1500
1000
500
0
15 w/w 0.1 mg/mL
30 w/w 0.05 mg/mL
30 w/w 0.1 mg/mL
30 w/w 0.2 mg/mL
E
CT2A GFP orthotopic transfection efficacy
GFP Concentration (pg/mL)
150
100
50
0
4-5-7 (0.2)
5-3-27 (0.2)
5-3-49 (0.2)
4-5-7 (0.1)
5-3-27 (0.1)
5-3-49 (0.1)

Figure S7. Flank and orthotopic transfection of IOMM-Lee and CT-2A tumors with PBAE nanoparticles.

(**A**) C57BL/6 mice were inoculated in the flank with 2M CT-2A cells, and athymic nude mice were inoculated with 2M IOMM-Lee cells. Delivery of 4-5-39 nanoparticle with luciferase exhibited luminescence signal at the 6 hour, 24 hour, and 48 hour mark. (**B**) Bioluminescence-based imaging of C57BL/6 mice implanted with CT-2A tumors in the flank and injected intratumorally with firefly-luciferase DNA-loaded 5-3-49 nanoparticle. The image shows successful transfection in four out of five mice using the in-vivo imaging system (IVIS) to detect emitted bioluminescence from the tumors (**C**) Humanized mice with double knock-out MHC I/II were inoculated on their flanks with 1M or 2M cells. IVIS imaging was performed 6 hours after nanoparticle injection to confirm luciferase signal. (**D**) Four different formulations of 4-5-39 nanoparticles with GFP reporter gene were injected into the intracranial IOMM-Lee tumors in athymic nude mice by convection enhanced delivery (CED). A GFP ELISA was performed to measure the transfection efficiency of each formulation. Each data bar represents median ± interquartile range with eight replicates. (**E**) Nanoparticle formulations with different GFP DNA loading concentrations have been used to achieve *in vivo* transfection in intracranially impanated CT-2A tumors in C57BL/6 mice. A GFP ELISA was performed to measure transfection efficiency of each candidate. Individual concentration measurements are represented on the graph along with the median and interquartile range for each group.

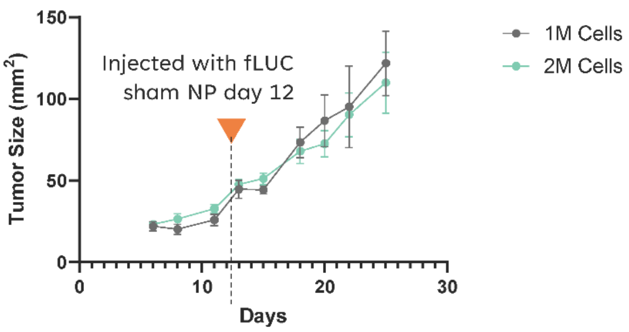

Figure S8: Pilot studies with IOMM-Lee cells in humanized mice to assess tumor growth. 1M and 2M IOMM-Lee cells were injected subcutaneously into the flanks of the humanized mice to observe tumor growth. When the tumors were 30 mm^2 on average (day 12), sham nanoparticles with luciferase DNA were injected intratumorally to see if nanoparticle presence would affect tumor growth.

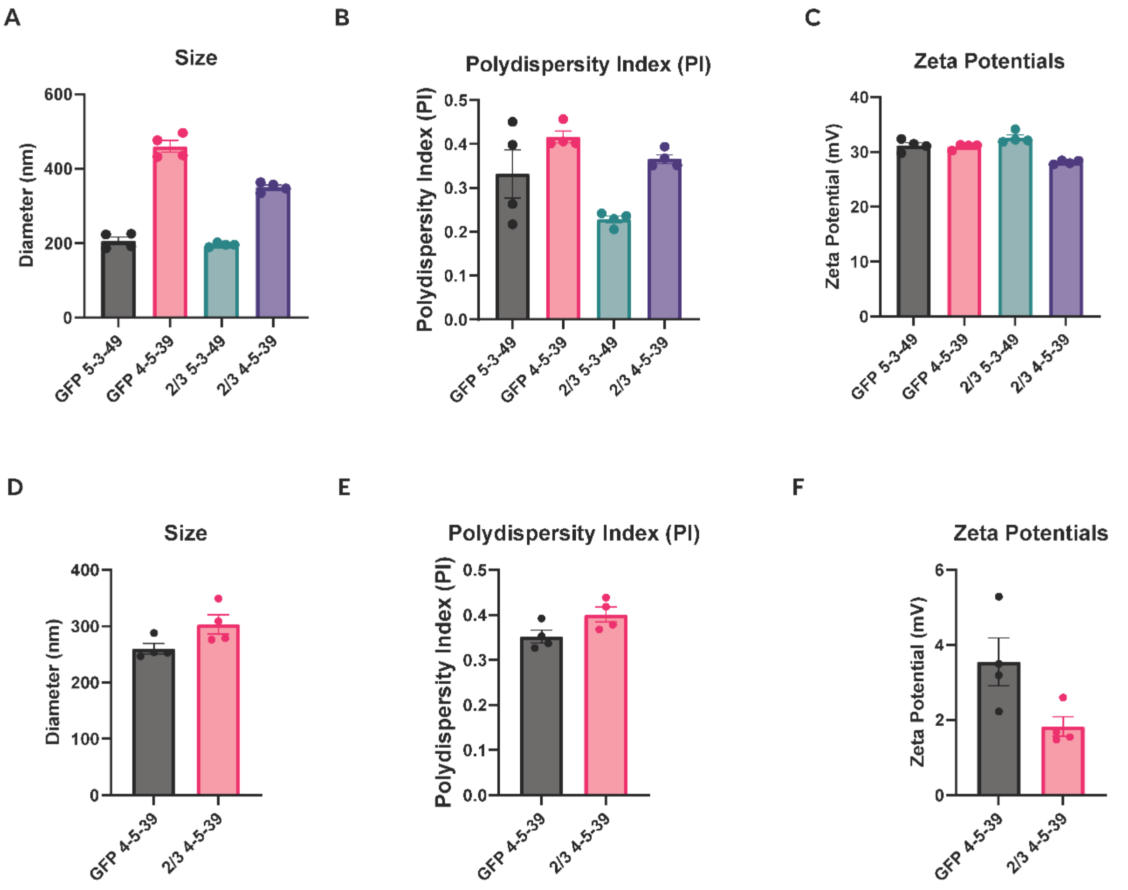

Figure S9. Characterization of *in vivo* nanoparticles. (**A** to **C**) Sizes, polydispersity indices, and zeta potentials of injected *in vivo* nanoparticles for the CT-2A studies were measured via DLS. The nanoparticles were formed at 15 w/w, 0.1 mg/mL DNA. (**D** to **F**) Sizes, polydispersity indices, and zeta potentials of injected *in vivo* nanoparticles for the IOMM-Lee humanized studies were measured via DLS. These nanoparticles were formulated at 30 w/w, 0.2 mg/mL DNA.

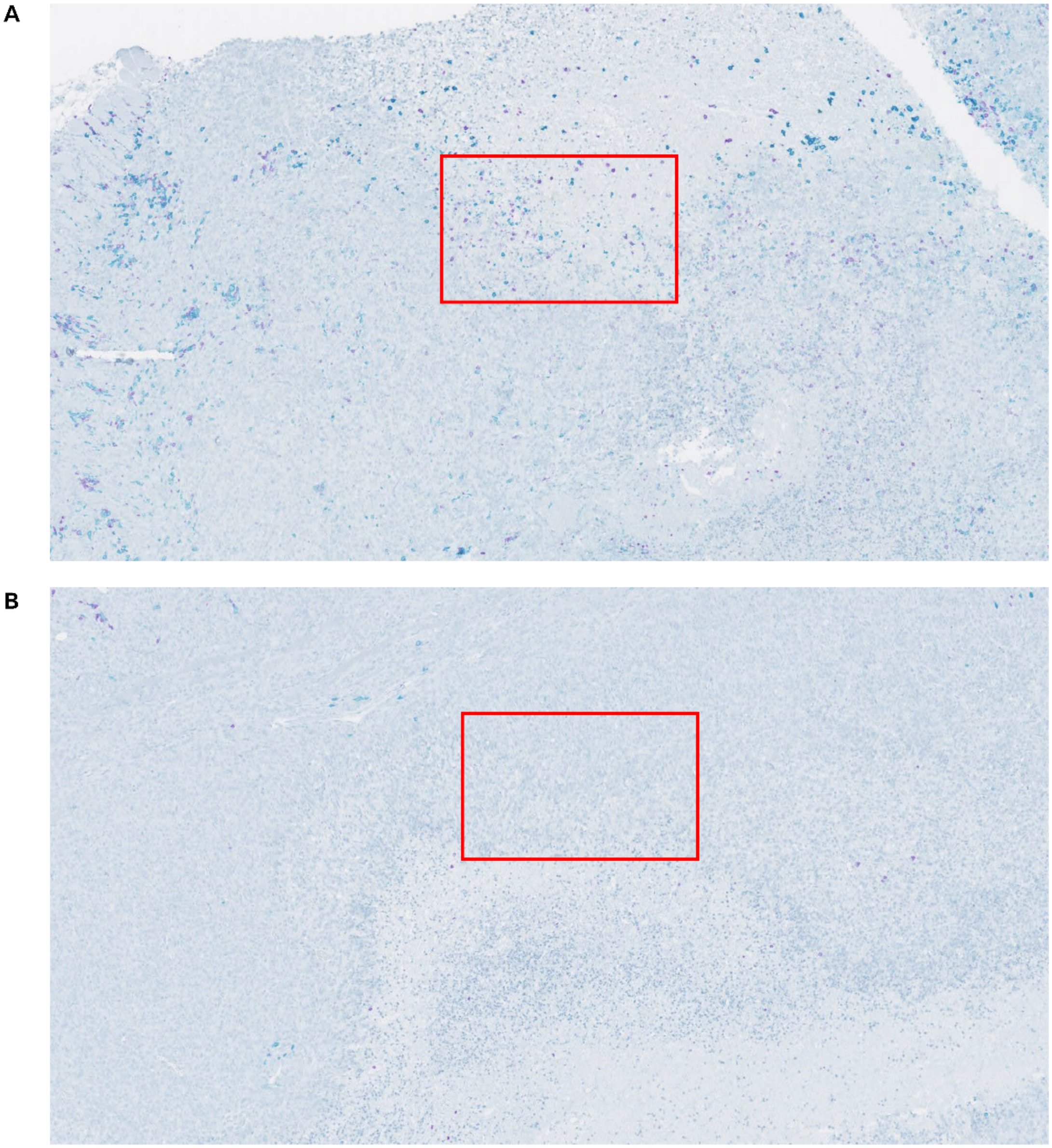

Figure S10. Meningioma tumor tissues IHC at 5X. (**A**) IOMM tumor IHC image for mouse treated with 4-1BBL and IL-12 NPs. (**B**) IOMM tumor IHC images for a mouse treated with luciferase NPs. FOXP3+ cells were stained with DAB, CD4+ cells were stained with teal, and CD8+ cells are stained with purple. More teal and purple cells are found in the mouse tumor treated with 4-1BBL and IL-12 NPs, illustrating T cell infiltration into the tumor. Red box indicates image shown in Figure 6.

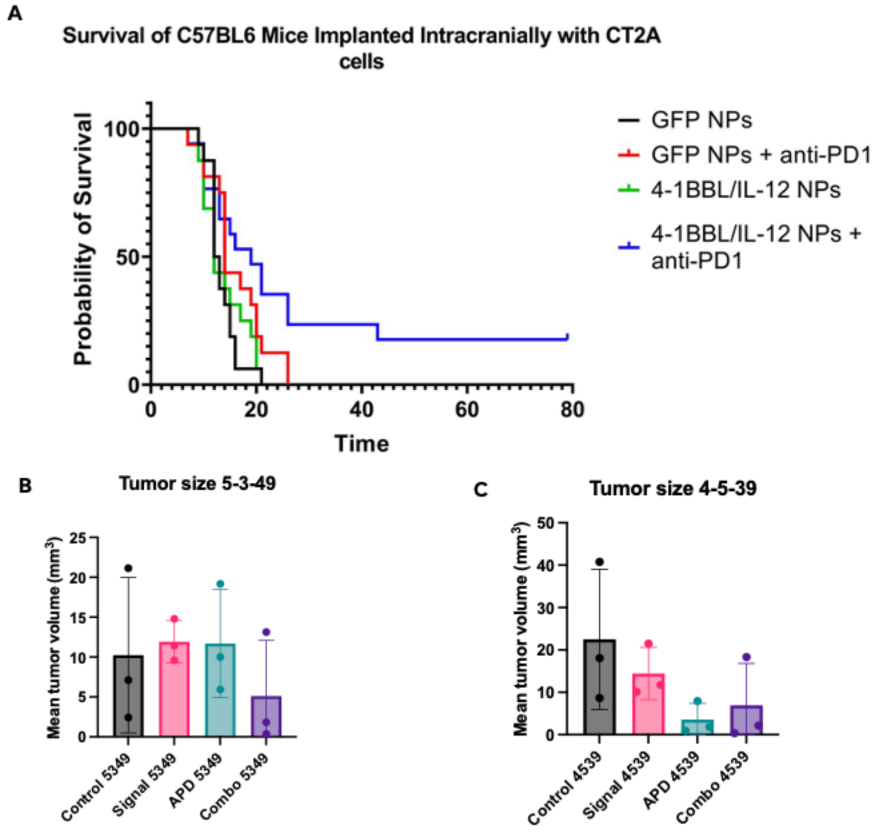

Figure S11. (A) Kaplan-Meier curve showing the survival of C57BL6 mice implanted with 125,000 firefly luciferase-tagged CT-2A glioma cells intracranially showing significant increase in overall survival of mice that received an intratumoral injection of the 4-1BBL/IL-12 nanoparticle (NP) and systemic anti-PD-1 therapy ($p= 0.003$, log-rank test) compared to the GFP nanoparticle group. (**B and C)** Tumor volumes based on H&E sections of the brains harvested from mice euthanized 72 hours after the final treatment in the 5-3-49 nanoparticle cohort (**B**) and 4-5-39 nanoparticle cohort (**C**) of mice implanted with 75,000 CT-2A glioma cells orthotopically.
